# Adult progenitor rejuvenation with embryonic factors

**DOI:** 10.1111/cpr.13459

**Published:** 2023-05-12

**Authors:** Peng Wang, Xupeng Liu, Yu Chen, Elwin Tan Jun‐Hao, Ziyue Yao, Jason Chua Min‐Wen, Benjamin Chua Yan‐Jiang, Shilin Ma, Wenwu Ma, Lanfang Luo, Luyao Guo, Dan Song, Ng Shyh‐Chang

**Affiliations:** ^1^ State Key Laboratory of Reproductive Biology, Institute of Zoology Chinese Academy of Sciences Beijing China; ^2^ Beijing Institute for Stem Cell and Regenerative Medicine Institute for Stem Cell and Regeneration, Chinese Academy of Sciences Beijing China; ^3^ University of Chinese Academy of Sciences Beijing China; ^4^ NUS Graduate School for Integrative Sciences and Engineering National University of Singapore Singapore Singapore; ^5^ Institute of Molecular and Cell Biology, Genome Institute of Singapore, Agency for Science Technology and Research Singapore Singapore; ^6^ Laboratory of Cancer Therapeutics, Program in Cancer and Stem Cell Biology, Duke‐National University of Singapore Medical School Singapore Singapore; ^7^ Laboratory of Cancer Epigenome, Division of Medical Science, National Cancer Centre Singapore Singapore Singapore

## Abstract

During ageing, adult stem cells' regenerative properties decline, as they undergo replicative senescence and lose both their proliferative and differentiation capacities. In contrast, embryonic and foetal progenitors typically possess heightened proliferative capacities and manifest a more robust regenerative response upon injury and transplantation, despite undergoing many rounds of mitosis. How embryonic and foetal progenitors delay senescence and maintain their proliferative and differentiation capacities after numerous rounds of mitosis, remains unknown. It is also unclear if defined embryonic factors can rejuvenate adult progenitors to confer extended proliferative and differentiation capacities, without reprogramming their lineage‐specific fates or inducing oncogenic transformation. Here, we report that a minimal combination of LIN28A, TERT, and sh‐p53 (LTS), all of which are tightly regulated and play important roles during embryonic development, can delay senescence in adult muscle progenitors. LTS muscle progenitors showed an extended proliferative capacity, maintained a normal karyotype, underwent myogenesis normally, and did not manifest tumorigenesis nor aberrations in lineage differentiation, even in late passages. LTS treatment promoted self‐renewal and rescued the pro‐senescence phenotype of aged cachexia patients' muscle progenitors, and promoted their engraftment for skeletal muscle regeneration in vivo. When we examined the mechanistic basis for LIN28A's role in the LTS minimum combo, *let‐7* microRNA suppression could not fully explain how LIN28A promoted muscle progenitor self‐renewal. Instead, LIN28A was promoting the translation of oxidative phosphorylation mRNAs in adult muscle progenitors to optimize mitochondrial reactive oxygen species (mtROS) and mitohormetic signalling. Optimized mtROS induced a variety of mitohormetic stress responses, including the hypoxic response for metabolic damage, the unfolded protein response for protein damage, and the p53 response for DNA damage. Perturbation of mtROS levels specifically abrogated the LIN28A‐driven hypoxic response in Hypoxia Inducible Factor‐1α (HIF1α) and glycolysis, and thus LTS progenitor self‐renewal, without affecting normal or TS progenitors. Our findings connect embryonically regulated factors to mitohormesis and progenitor rejuvenation, with implications for ageing‐related muscle degeneration.

## Graphical abstract

Model of how LIN28A, mitochondrial reactive oxygen species, and the HIF1α‐associated metabolic stress response network promotes stem cell self‐renewal.

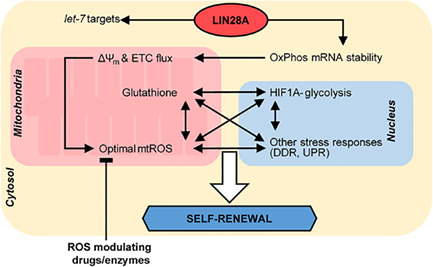

## 1 INTRODUCTION

The regenerative properties of muscle stem cells decline with ageing as they enter an irreversibly senescent state, thereby failing to proliferate or differentiate, with important implications for transplantation and regenerative medicine.^1^ The regenerative capacity of skeletal muscles depends on muscle stem cells and muscle progenitors, which proliferate in response to tissue damage, and which either fuse and differentiate to regenerate myofibres or self‐renew to restore the pool of stem cells.^2^, ^3^, ^4^, ^5^ In contrast to aged animals, whose pro‐senescent stem cells fail to proliferate and differentiate properly in response to injury, juvenile animals are able to manifest more robust regenerative responses in general.^6^, ^7^, ^8^ The conserved relationship between juvenility and tissue regeneration was first discussed by Darwin et al.,^9^, ^10^ but the precise mechanisms that underlie juvenility and rejuvenation had remained unclear.

Skeletal muscles constitute ~40% of the young human body mass. Sarcopenia is the gradual decline of skeletal muscle mass and function with ageing. With ageing, muscles manifest a profound regenerative defect that contributes to elderly frailty in sarcopenia and cachexia. Both changes in the extrinsic microenvironment and stem cell‐intrinsic mechanisms may contribute to this regenerative decline.^2^, ^11^, ^12^ Recent studies have demonstrated that both the numbers and the functionality of adult muscle stem cells decline with ageing, especially after geroconversion and senescence.^13^, ^14^, ^15^, ^16^, ^17^, ^18^ In contrast, embryonic, foetal and perinatal muscle progenitors are widely known to possess extended proliferative capacities, compared with adult muscle progenitors, which in turn possess higher proliferative capacities than aged adult muscle progenitors.^19^, ^20^, ^21^, ^22^ This is despite the fact that embryonic and perinatal progenitors also sustained numerous rounds of mitosis during embryogenesis and foetal development. For instance, it is known that Pax3+ muscle progenitors give rise to all embryonic, foetal, and adult myoblasts and myofibres.^3^, ^23^, ^24^, ^25^ While it is widely known that muscle progenitors' lifespan inexorably decline with development and ageing, the molecular principles responsible for this phenomenon have remained incompletely understood. Thus we asked, what embryonically regulated factors change with adult human muscle progenitor ageing, and what is the minimal set of embryonically regulated factors that are needed to delay the pro‐senescence trend in adult human muscle progenitors?

The mammalian p53 transcription factor family comprises three members p53, p63, and p73. The transcription factors evolved from an ancestral *p63/p73* gene that can be found in most invertebrates,^26^, ^27^ mediating stress responses upon DNA damage. Although the full‐length isoforms p53α, TAp63α, and TAp73α all function as tumour suppressors in adulthood,^28^, ^29^ they also play important roles during embryogenesis. While p63 governs epithelial progenitor cells and epidermis development,^30^, ^31^ p73 functions in neuronal development and multi‐ciliated cell differentiation.^32^ The p53 factor is not only finely regulated in expression during embryogenesis,^33^, ^34^ but also functions in neural tube closure^35^ and craniofacial skeletal, neuronal, and muscle tissue development.^36^ Moreover, the dosage of p53 is also tightly regulated by the Mdm2/Mdm4 family of E3 ubiquitin ligases and the *miR‐125/lin‐4* family of microRNAs, to facilitate normal tissue development.^37^, ^38^, ^39^ Indeed, previous studies had shown that p53 is important in regulating many tissue progenitors' self‐renewal and differentiation.^40^, ^41^, ^42^

Two other factors that are tightly regulated during embryonic development in multiple tissue lineages, are LIN28 and TERT, both of which are RNA‐binding proteins that are typically only highly expressed in embryonic progenitors in mammals.^43^, ^44^, ^45^ Here, by examining a series of molecular markers associated with senescence in our ageing adult muscle progenitors and performing a mini‐screen for embryonic factors, we found that a minimal combination of LIN28A, hTERT, and p53 shRNA (LTS) could dramatically delay the senescence of human muscle progenitors. LTS (but not TS) factors could dedifferentiate adult muscle progenitors and improve their regenerative potential in vivo. Mechanistically, we found that LIN28A collaborated with TS to optimize mitochondrial reactive oxygen species (mtROS) and stress‐responsive signalling pathways, thereby inducing mitohormesis and restoring juvenility. Perturbation of mtROS levels specifically abrogated the LIN28A‐driven hypoxic response in Hypoxia Inducible Factor‐1α (HIF1α) and glycolysis, and thus LTS progenitor self‐renewal, without affecting normal or TS progenitors. Our findings connect embryonic factors to mitohormesis during the process of muscle progenitor self‐renewal and rejuvenation, with implications for ageing‐related muscle degeneration in cachexia and sarcopenia.

## 2 RESULTS

### 2.1 LTS factors delay adult progenitor senescence without transformation while permitting normal differentiation

**FIGURE 1:**
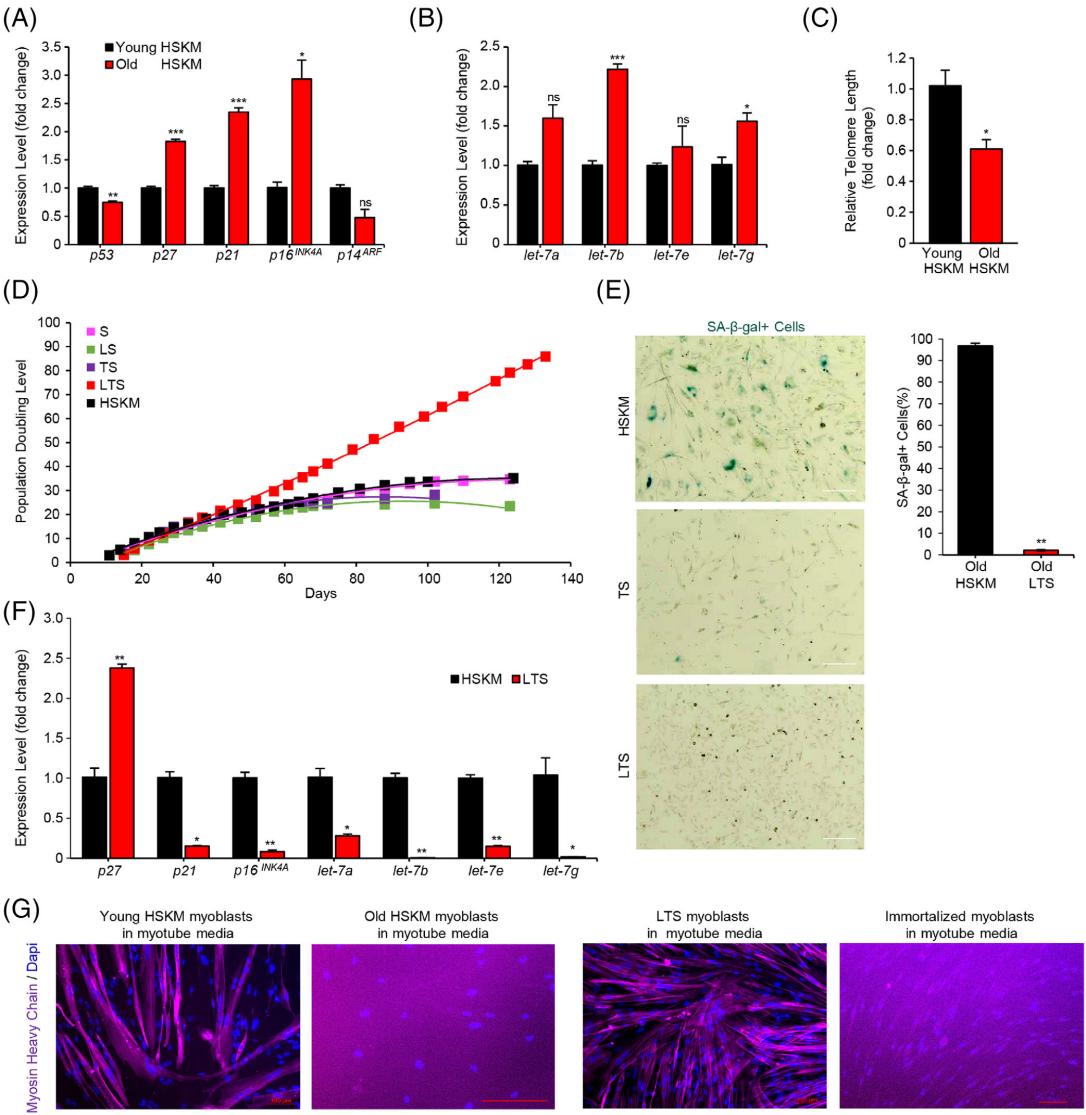
The effects of LIN28A, TERT, and sh‐p53 (LTS) factors on adult human muscle progenitor senescence and differentiation. (A) Quantitative Reverse Transcription‐Polymerase Chain Reaction (RT‐PCR) for mRNAs of cell cycle inhibitors in aged adult human skeletal muscle (HSKM) myoblasts, relative to young adult HSKM myoblasts (*N* = 3 wells of cells for each group). (B) Quantitative RT‐PCR for *let‐7* microRNAs in aged adult HSKM myoblasts(red columns), relative to young adult HSKM myoblasts (black columns; *N* = 3 wells of cells for each group). (C) Quantitative RT‐PCR for telomere length in aged adult HSKM myoblasts, relative to young adult HSKM myoblasts (*N* = 3 wells of cells for each group). (D) Population doubling curves for young HSKM myoblasts (black), and young adult HSKM myoblasts transduced with viral sh‐p53 (S, pink), and LIN28A (LS, green), or hTERT (TS, purple), or LIN28A and hTERT (LTS, red). While young adult HSKM and other transgenic myoblasts started to undergo senescence by 60 days, before the 30th population doubling, the LTS myoblasts (red) continued to proliferate steadily beyond 120 days and beyond the 90th population doubling. (E) Brightfield micrographs of senescence‐associated β‐galactosidase positive (SA‐β‐gal+) cells in 100‐day‐old adult HSKM myoblasts and 100‐day‐old LTS myoblasts. Quantification of senescence‐associated β‐galactosidase positive (SA‐β‐gal+) cells in 100‐day‐old adult HSKM myoblasts, relative to 100‐day‐old LTS myoblasts (*N* = 3 wells of cells for each group). (F) Quantitative RT‐PCR for *p27* ^*KIP1*^, *p21* ^*CIP1*^, *p16* ^*INK4A*^, and *let‐7* microRNAs in LTS myoblasts, relative to young adult HSKM myoblasts (*N* = 3 wells of cells for each group). (G) Immunofluorescence staining for the myotube protein marker myosin heavy chain in young HSKM and LTS myoblasts, relative to old HSKM myoblasts and immortalized (hTERT‐CyclinD1‐CDK4^R24C^) myoblasts, that were cultured in myogenic differentiation media. Cells were counterstained with 4'6‐Diamidino‐2‐Phenylindole to visualize the myonuclei. Scale bars 100 μm. *p < 0.05, **p < 0.01, ***p < 0.001; ns, not significant.

To address this question, we used young and old primary adult human skeletal muscle (HSKM) progenitors to screen for a variety of embryonically regulated factors that are not lineage‐specific, to biomimic the foetal growth phase. We defined ‘young’ as <5 population doublings, and ‘old’ as more than 20 population doublings, based on prior experience with replicative senescence.^46^ First, we found that old progenitors, compared with progenitors, showed significantly higher levels of cell cycle inhibitors such as *p21* ^*WAF1*^, *p27* ^*KIP1*^, and *p16* ^*INK4a*^ (Figure 1A). Furthermore, the anti‐proliferative family of *let‐7* microRNAs, especially *let‐7b* and *let‐7g*, also accumulated to higher levels in old progenitors, compared with young progenitors (Figure 1B), consistent with previous studies of primary human muscle samples.^47^ In contrast, the telomeres of old progenitors were significantly shorter than young progenitors (Figure 1C). Although the mRNA expression levels of *p53* and *p14* ^*ARF*^ trended towards downregulation, we found that p53 protein was accumulating to higher levels in old progenitors (Figures S1 and S2). In contrast, we had found that the p53 inhibitor *Mdm4* and the telomerase complex component *Tep1* were amongst the most highly upregulated genes in mouse Lin28a + MuSCs (Preprint).^48^ These preliminary results spurred us to test some embryonically regulated factors that are not lineage specific, to attempt to prevent or delay the pro‐senescence trend. We tested LIN28A to regulate the accumulating *let‐7* miRNAs and other developmental genes, hTERT to lengthen and restore the telomeres, and short hairpin RNAs (shRNAs) against p53 (which also transactivates *p21* ^*WAF1*^), *p16* ^*INK4a*^, *p14* ^*ARF*^, or *Rb*, all of which are tightly regulated in embryogenesis and accumulate with senescence.^40^, ^42^, ^49^, ^50^, ^51^, ^52^ In contrast, we omitted screening any mutant oncogenes which overcome existing senescence, instead of delay senescence. Oncogenes which overcome (instead of delay) senescence might cause irreversible transformation, aberrant differentiation of progenitors,^46^ and thus tumour growth. We also omitted screening pluripotency factors, such as OCT4, SOX2, NANOG, and so forth, because they are lineage‐specific to pluripotent stem cells, and we found that none of the pluripotency markers were increased in mouse Lin28a + muscle stem cells (Preprint).^48^

Our results showed that young progenitors quickly undergo replicative senescence, plateauing sigmoidally at ~30 population doublings, and no single factor alone could prevent the senescence (Figures 1D and S3). However, when LIN28A was combined with hTERT and a shRNA against *TP53* (LTS; Figure S4), human muscle progenitors could self‐renew and proliferate beyond 90 population doublings, with a linear population doubling curve up till ~206 population doublings. Furthermore, each factor was necessary in the LTS combo, as lacking any one of the three factors resulted in either senescence (Figure 1D) or apoptosis (data not shown).

When 100‐day‐old control progenitors and 100‐day‐old LTS progenitors were examined for senescence‐associated β‐galactosidase (SA‐β‐gal) staining, 96.7% of control progenitors were SA‐β‐gal^+^, whereas only 2.2% of LTS progenitors were SA‐β‐gal^+^, indicating that the LTS combo was profoundly protective against senescence (Figure 1E). When examined in detail, we found that the LTS combo did prevent the ageing‐induced accumulation of *p21* ^*WAF1*^, *p16* ^*INK4a*^, and *let‐7* microRNAs (Figure 1F), while restoring the telomeres (Figure S5). Transcriptomic profiling further confirmed that many senescence markers such as *CDKN1A*, *CDKN1C*, *CDKN2A*, and *TP53*, were significantly reduced, though *CDKN2B* was slightly increased, whereas pluripotency markers such as *OCT4*, *SOX2*, *NANOG*, *KLF4*, *KLF5*, *ESRRB*, and the *SIRT1‐7* sirtuin family members were all not upregulated in LTS progenitors (Figure S6).

We then tested if the LTS progenitors could still differentiate properly. We expect oncogene‐transformed progenitors to be unable to differentiate properly, since progenitors need to completely withdraw from the cell cycle *and* properly activate epigenetic remodelling before they can undergo terminal differentiation. Surprisingly, we found that the rejuvenated 100‐day‐old LTS progenitors could still robustly differentiate into myosin heavy chain (MHC)^+^, α‐actinin^+^, myogenin^+^ multinucleated myotubes, just like young HSKM progenitors (Figures 1G and S7).

In contrast, the immortalized progenitors harbouring oncogenic CDK4^R24C^, cyclin D1, and hTERT failed to differentiate properly, and only expressed very weak levels of MHC, with few multinucleate myotubes (Figure 1G). Similarly, the 100‐day‐old senescent progenitors could not differentiate into multinucleated myotubes (Figure 1G). We also tested if LTS progenitors were reprogrammed into primitive mesenchymal or mesodermal progenitors by subjecting them to adipogenic, osteogenic, and chondrogenic differentiation conditions. We did not detect significant adipogenesis, osteogenesis, or chondrogenesis (data not shown). We also profiled LTS cells for their mRNA expression of myogenic markers. LTS myotubes were only mildly weaker in some myogenic markers (*ACTA1*, *MYH1*, *MYH8*), but also mildly stronger in several other myogenic markers (*MYF5*, *MYOD*, *MYH7*), while maintaining similar expression in several myogenic markers (*MYOG*, *MYH3*) compared with young adult HSKM myotubes (Figure S7). These results indicate that LTS progenitors can properly activate a normal differentiation programme.

Secondly, we tested if the LTS factors would lead to genomic instability, given the partial inhibition of *TP53* by RNAi. When we subjected 100‐day‐old LTS progenitors to chromosomal analysis, the highly passaged cells still displayed a normal diploid karyotype, similar to highly passaged ES cells (Figure S8). In contrast, 100‐day‐old TS progenitors displayed a variety of aneuploid karyotypes, including the loss of numerous chromosomes, and the appearance of dicentric, ring, and marker chromosomes (Figure S8). We also performed immunofluorescence staining for the expression of a series of DNA damage markers in LTS progenitors, and found that the expression of PARP‐1, XRCC1, γ‐H2A.X, and 53BP1 in LTS progenitors was relatively lower than in TS progenitors (Figure S9A). These results indicate that LTS progenitors can properly activate a normal DNA repair and self‐renewal programme.

Thirdly, subcutaneous transplantation of LTS progenitors with Matrigel into immunodeficient NOD/SCID/Il2rγ^−/−^ (NSG) mice further demonstrated that oncogenic transformation did not occur. Luciferase‐labelled LTS progenitors gradually declined and disappeared within 2 weeks of subcutaneous transplantation (Figure S9B). In comparison, all other transformed cancer cell lines form tumours upon subcutaneous transplantation in NSG mice. These results suggested that LIN28A could ensure proper self‐renewal with normal differentiation potential and genomic stability, and thus prevent tumorigenicity.

### 2.2 LTS factors can rejuvenate aged muscle progenitors for transplantation

**FIGURE 2:**
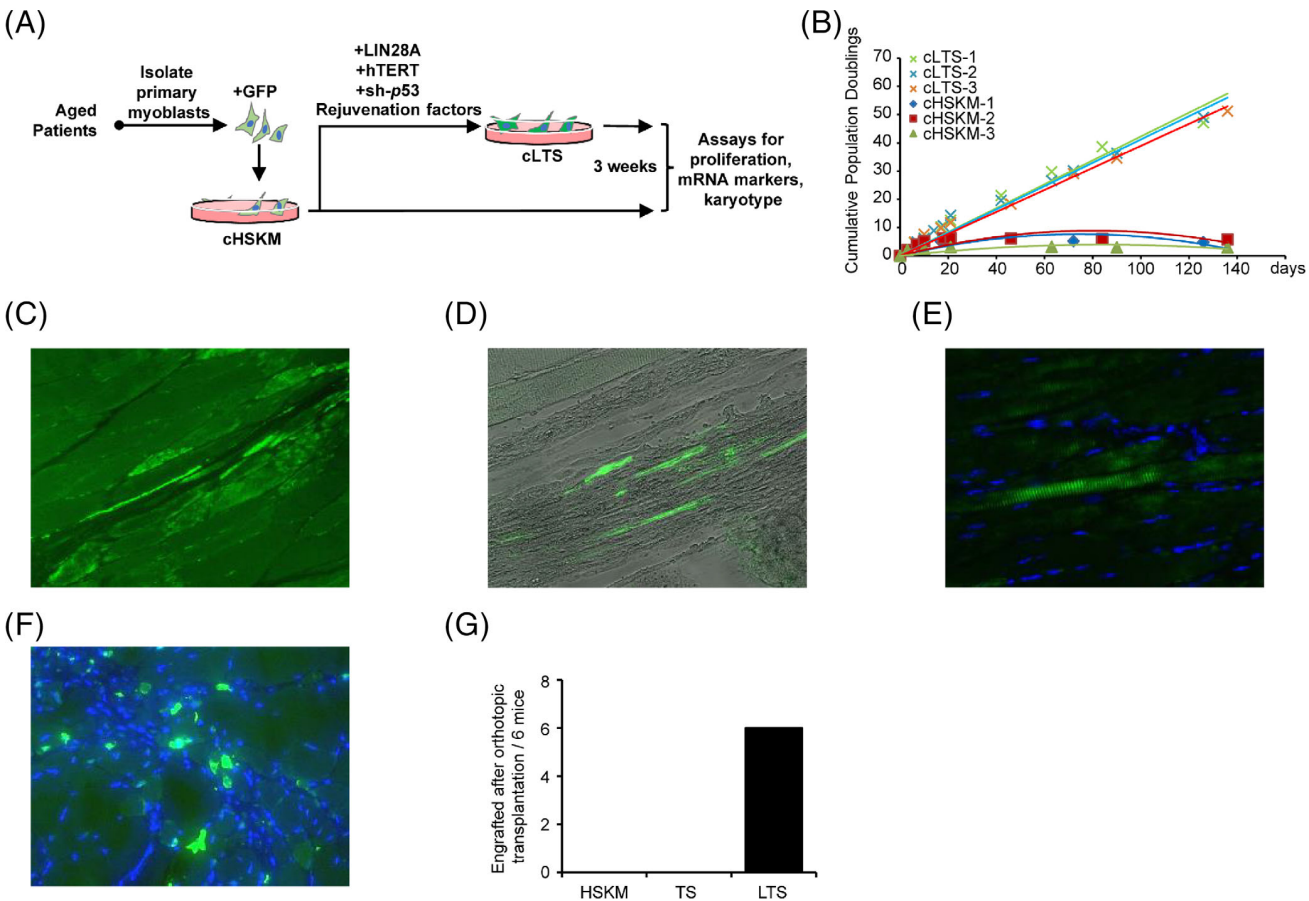
LIN28A, TERT, and sh‐p53 (LTS) factors rejuvenate aged muscle progenitors for transplantation in vivo. (A) Schematic for derivation and rejuvenation of aged cachexia patients' myoblasts. (B) Population doubling curves for three representative aged cachexia patients' human skeletal muscle (HSKM) myoblasts (cHSKM‐1/2/3), and their corresponding LTS‐treated lines (cLTS‐1/2/3). (C–F) Immunofluorescence staining for GFP showed engraftment of LTS‐treated aged progenitors, after orthotopic injection of 1 × 10^6^ cells into the cryoinjured tibialis anterior (TA) muscles of NOD/SCID/Il2rγ^−/−^ (NSG) mice. (C). GFP+ LTS progenitors differentiated into elongated myofibres, or (E) fused with mouse myofibres to form GFP+ domains within the myofibres, and (F) some persisted as self‐renewing progenitors on the periphery of the myofibres. (G) Quantification of NSG mice that showed engraftment into cryoinjured TA muscles after orthotopic transplantation of 1 × 10^6^ HSKM, TS, and LTS progenitors (*N* = 6 mice for each group).

To test the utility of the LTS factors in aged muscle progenitors of elderly patients, we transduced aged muscle progenitors from elderly cachexia patients. We previously found that cachexia can lead to suppression of myocyte growth.^53^ When we cultured aged patients' HSKM progenitors, we also found that they rapidly underwent senescence within 6 population doublings (Figures 2A,B), which is significantly less than healthy young adult muscle progenitors (~30 population doublings, Figure 1B). However, upon treatment with the LTS factors (Figure 2A), the aged HSKM progenitors successfully delayed their rapid senescence and reset their ageing clocks, with linear population doubling curves up till ~200 population doublings (Figure 2B).

**FIGURE 3:**
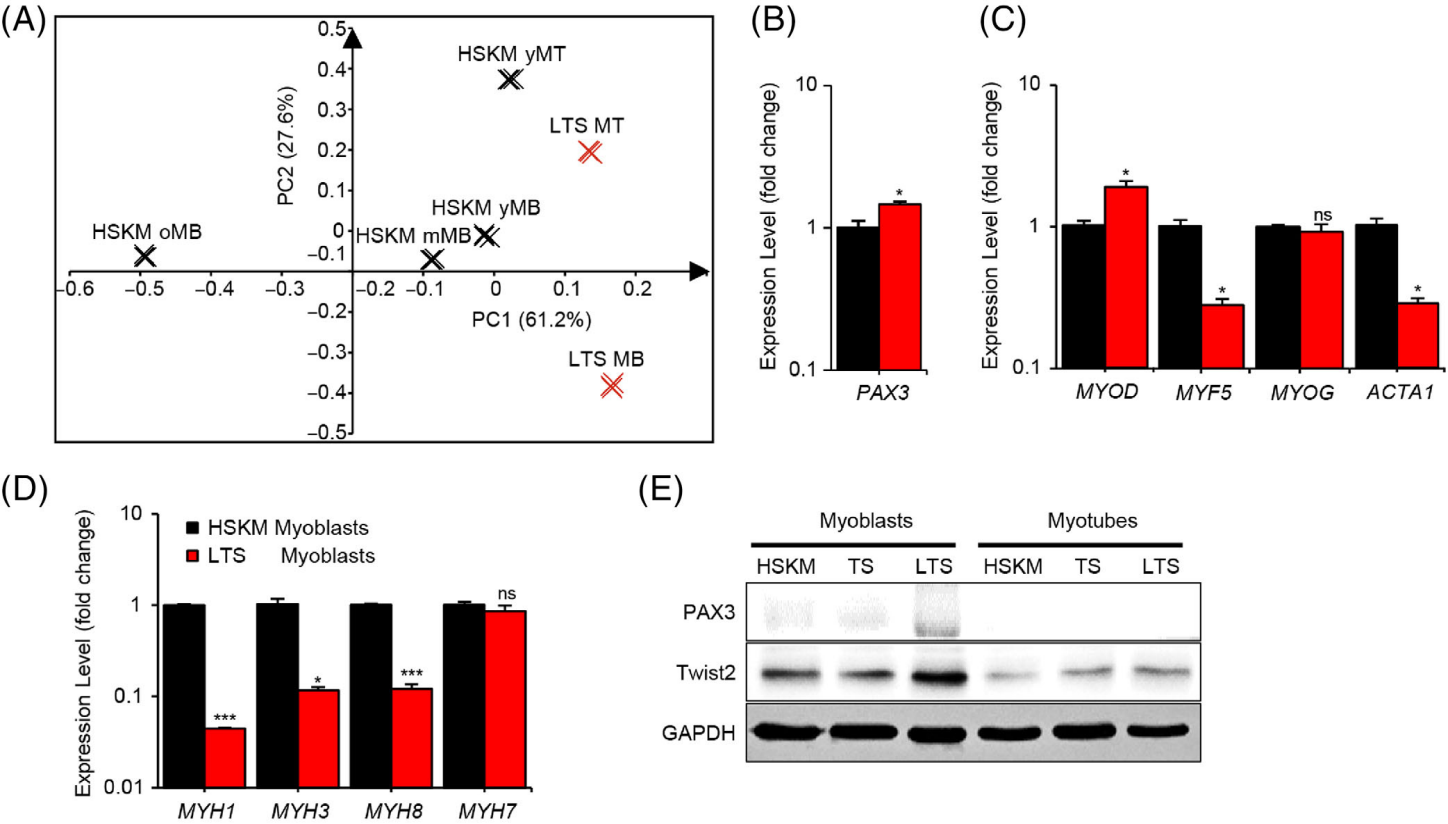
LIN28A, TERT, and sh‐p53 (LTS) progenitors are less aged and less differentiated than primary adult human progenitors. (A) Principal components analysis of transcriptomic data for LTS myoblasts and myotubes (red), relative to human skeletal muscle (HSKM) young, intermediate, and old myoblasts, and young HSKM myotubes (black). (B,C) Quantitative Reverse Transcription‐Polymerase Chain Reaction (RT‐PCR) for *PAX3*, *MYOD*, *MYF5*, *MYOG*, and *ACTA1* in LTS myoblasts, relative to young adult HSKM myoblasts (*N* = 3 wells of cells for each group). (D) Quantitative RT‐PCR for the terminal differentiation markers *MYH1*, *MYH3*, *MYH8*, and *MYH7* in LTS myoblasts, relative to young adult HSKM myoblasts (*N* = 3 wells of cells for each group). (E) Western blot for PAX3, TWIST2 protein, relative to GAPDH protein, in young HSKM, TS, and LTS myoblasts and myotubes. The quantification of WB bands is shown in Figure S2. *p < 0.05, ***p < 0.001; ns, not significant.

To test if LTS treatment can produce engraftable stem cells for cell therapy, LTS‐aged progenitors were massively expanded, whereas control HSKM progenitors were pooled together. In contrast to subcutaneous transplantation, orthotopic transplantation was successful, as expected of functional MuSCs (Figure 2C–G). 1 × 10^6^ GFP+ LTS and control progenitors were injected orthotopically into the tibialis anterior (TA) muscles of NSG mice after cryoinjury. Immunofluorescence staining for GFP showed that there was significant engraftment of LTS cachectic progenitors 30 days after injection (Figure 2C–F), whereas little engraftment of GFP^+^ control progenitors was observed in the regenerating muscles. The GFP^+^ LTS human progenitors had mostly differentiated into elongated myofibres (Figure 2C,D), or fused with mouse myofibres to form GFP^+^ domains within the myofibres (Figures 2C–E), whilst a minority persisted as self‐renewing human progenitors on the periphery of the myofibres (Figure 2F). In contrast, GFP+ HSKM and TS progenitors could never engraft after orthotopic transplantation (Figure 2G), as these myoblasts have committed and differentiated into PAX3^low^ cells (Figure 3E). However, LTS progenitors can reactivate PAX3 protein expression, engraft, and contribute to muscle regeneration in vivo like normal MuSCs, without forming tumours even after 12 months (Figure 3E). Overall, LTS treatment dramatically increased the engraftment efficiency of human muscle progenitors (Figure 2G), which is typically very low.^54^, ^55^ Thus, even aged muscle progenitors' regenerative functions can be restored with LTS treatment.

### 2.3 LTS progenitors are less aged and differentiated than primary progenitors

We compared LTS with normal primary HSKM cells, both myoblasts and their derivative myotubes, and both young and old, using transcriptomic profiling to assess their cellular states. Principal components analysis (PCA) of the mRNA profiles revealed that the different cell types were clearly separated by their senescence and differentiation states (Figure 3A). The differentiation vector, defined as the line of best fit between HSKM myoblasts and myotubes in the dimensionally reduced 2D gene expression space, was approximately parallel to the LTS cells' differentiation vector, suggesting again that the differentiation programme was preserved after LTS treatment. The senescence vector, defined as the line of best fit between the HSKM young, intermediate, and old myoblasts in the dimensionally reduced 2D gene expression space, was approximately orthogonal to the differentiation vector, suggesting that terminal differentiation and senescence are two distinct processes even if both processes involved a cell cycle exit. Based on the differentiation and senescence vectors defined by the primary HSKM cells, LTS myoblasts appeared dedifferentiated and more juvenile, compared with young adult myoblasts (Figure 3A).

To confirm this observation, we performed qRT‐PCR for a variety of myogenic markers to assess the differentiation status of young adult HSKM and LTS progenitors. Our results showed that LTS progenitors were significantly higher in expression of *PAX3*, an embryonic myogenic transcription factor, than in HSKM progenitors (Figure 3B). While the myoblast commitment factor *MYOD* was higher (Figure 3C), the myogenic determination factor *MYF5* was lower (Figure 3C), and the myogenic differentiation factor myogenin *MYOG* remained similarly low (Figure 3C). *MYOD* and *MYF5* are homologous in sequence and co‐belong to the MRF family, thus they have functionally overlapping roles in promoting myoblast specification.^56^ Because in LTS muscle progenitors, both *MYOD* and the muscle stem cell factor *PAX3* are already highly expressed in LTS, it is possible that *MYF5* was slightly decreased in expression as a compensatory response. Most other myogenic differentiation markers were even lower in LTS progenitors than in HSKM progenitors, including skeletal muscle actin A1 (*ACTA1*; Figure 3C), and several MHC isoforms (Figure 3D). These results indicated again that LTS myoblasts were dedifferentiated. In contrast, in the LTS myotubes, the expression of *MYF5* and *MYOD*, as well as *MYOG* and *MYH3* are all slightly enhanced, whereas some myogenic markers are slightly lower. Overall, we believe these differences are too slight to be biologically significant, and the myogenic differentiation capacity of LTS cells can be considered similar to HSKM cells (Figure S7).

We also performed western blots for PAX3 and TWIST2 protein expression in LTS, TS and HSKM cells. LTS progenitors showed the highest PAX3 protein levels, compared with HSKM and TS progenitors (Figures 3E and S2). All the progenitors showed significant declines in PAX3 and TWIST2 protein levels, as they differentiated into myotubes (Figures 3E and S2), and as expected of myogenesis.^57^ The increased PAX3 protein, an embryonic myogenic transcription factor, supports the transcriptomic observations on LTS progenitors.

### 2.4 LIN28A aids rejuvenation via *let‐7*‐independent effects on mRNA expression

**FIGURE 4:**
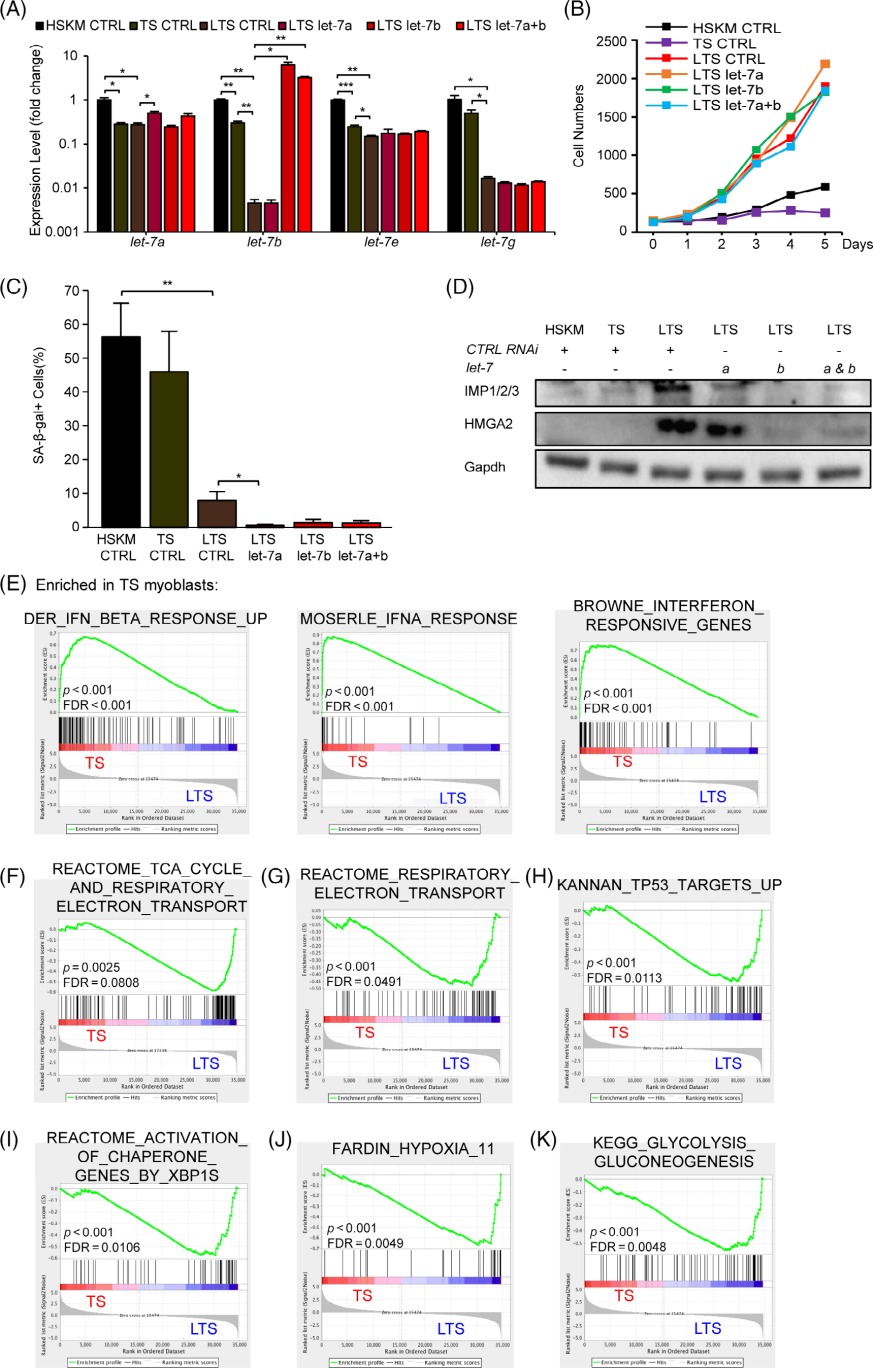
*Let‐7* repression is insufficient to explain LIN28A, TERT, and sh‐p53 (LTS) rejuvenation of adult muscle progenitors. (A) Quantitative RT‐PCR for *let‐7* microRNAs in TS and LTS myoblasts, relative to young adult human skeletal muscle (HSKM) myoblasts, after control (CTRL), *let‐*7a, *let‐7b*, or combined *let‐*7a and *let‐7b* (let‐7a + b) mimic transfection (*N* = 3 wells of cells for each group). (B) Cell counts after control RNAi (CTRL), *let‐7*a, *let‐7*b, or combined *let‐7*a and *let‐7*b (*let‐7*a + b) mimic transfection in equal initial numbers of TS and LTS myoblasts, relative to young adult HSKM myoblasts (*N* = 3 wells of cells for each group). (C) Quantification of senescence‐associated β‐galactosidase (SA‐β‐gal)+ cells in TS and LTS myoblasts, relative to young adult HSKM myoblasts, after control RNAi (Ctrl RNAi), *let‐*7a, *let‐7b*, or combined *let‐*7a and *let‐7b* (let‐7a + b) mimic transfection (*N* = 5 wells of cells for each group). (D) Western blot analysis for the *let‐7* targets IGF2BP1/2/3 (IMP1/2/3) and HMGA2 protein, relative to Gapdh protein, in young adult HSKM, TS, and LTS myoblasts, after control (CTRL), *let‐*7a (a), *let‐7b* (b), or combined *let‐*7a and *let‐7b* (A,B) mimic transfection. The quantification of WB bands is shown in Figure S2. (E) Representative gene set enrichment analysis (GSEA) enrichment profiles for RNAseq analysis of TS myoblasts. (F–K) Representative GSEA enrichment profiles for RNAseq analysis of LTS myoblasts. *p < 0.05, **p < 0.01, ***p < 0.001.

To dissect the mechanism for LIN28A's rejuvenating effect, we returned to primary human progenitors cultured ex vivo. Our RNA profiling studies earlier had shown that *let‐7* family members accumulated to higher levels in ageing progenitors (Figure 1B). Both TS and LTS transduction lowered the *let‐7* levels, with LIN28A further suppressing *let‐7b*, *let‐7e*, and *let‐7g* levels (Figure 4A). To test if LIN28A's rejuvenation effect in muscle progenitors was mediated by *let‐7*, we transfected mature *let‐7* mimics in an attempt to abrogate transgenic LIN28A's effect on proliferation (Figure 4A). However, we found that neither *let‐7a*, nor *let‐7b*, nor both, had any effect on LTS progenitors' proliferation rate (Figure 4B). Moreover, *let‐7* overexpression also failed to increase the numbers of senescent SA‐β‐gal^+^ cells (Figure 4C), even though important *let‐7* targets such as IMP1/2/3 and HMGA2^45^ had been almost completely depleted by the *let‐7* overexpression (Figures 4D and S2). These results suggest that *let‐7* repression alone cannot fully explain LIN28A's effect on muscle progenitor self‐renewal.

Besides regulating the *let‐7* microRNAs, Lin28a also binds and regulates mRNAs. Thus, we analysed the transcriptomic profiles of LTS progenitors, and compared them against TS progenitors by gene set enrichment analysis (GSEA). The top downregulated signatures in LTS progenitors were almost completely dominated by response targets (Figure 4E). This is consistent with findings that an interferon‐driven innate immune or inflammageing response is activated during senescence,^58^ and further corroborated the senescence of TS progenitors, which the addition of LIN28A prevented. Our analysis further revealed that LIN28A's upregulated signatures were primarily metabolic in nature, consisting of mitochondrial oxidative phosphorylation (OxPhos) genes (Figure 4F,G), stress‐responsive signatures such as the unfolded protein response (UPR) mediated by XBP1s activity (Figure 4H,I), and the hypoxic stress response and glycolysis targets mediated by HIF1α (Figure 4J,K). Interestingly, the HIF1α‐mediated hypoxic stress response was also observed in mouse Lin28a + MuSCs (Preprint),^48^ indicating that Lin28a's induction of HIF1α is a conserved mechanism in both adult human muscle progenitors and mouse MuSCs.

### 2.5 LIN28A optimizes OxPhos and mtROS to induce HIF1α‐glycolysis

**FIGURE 5:**
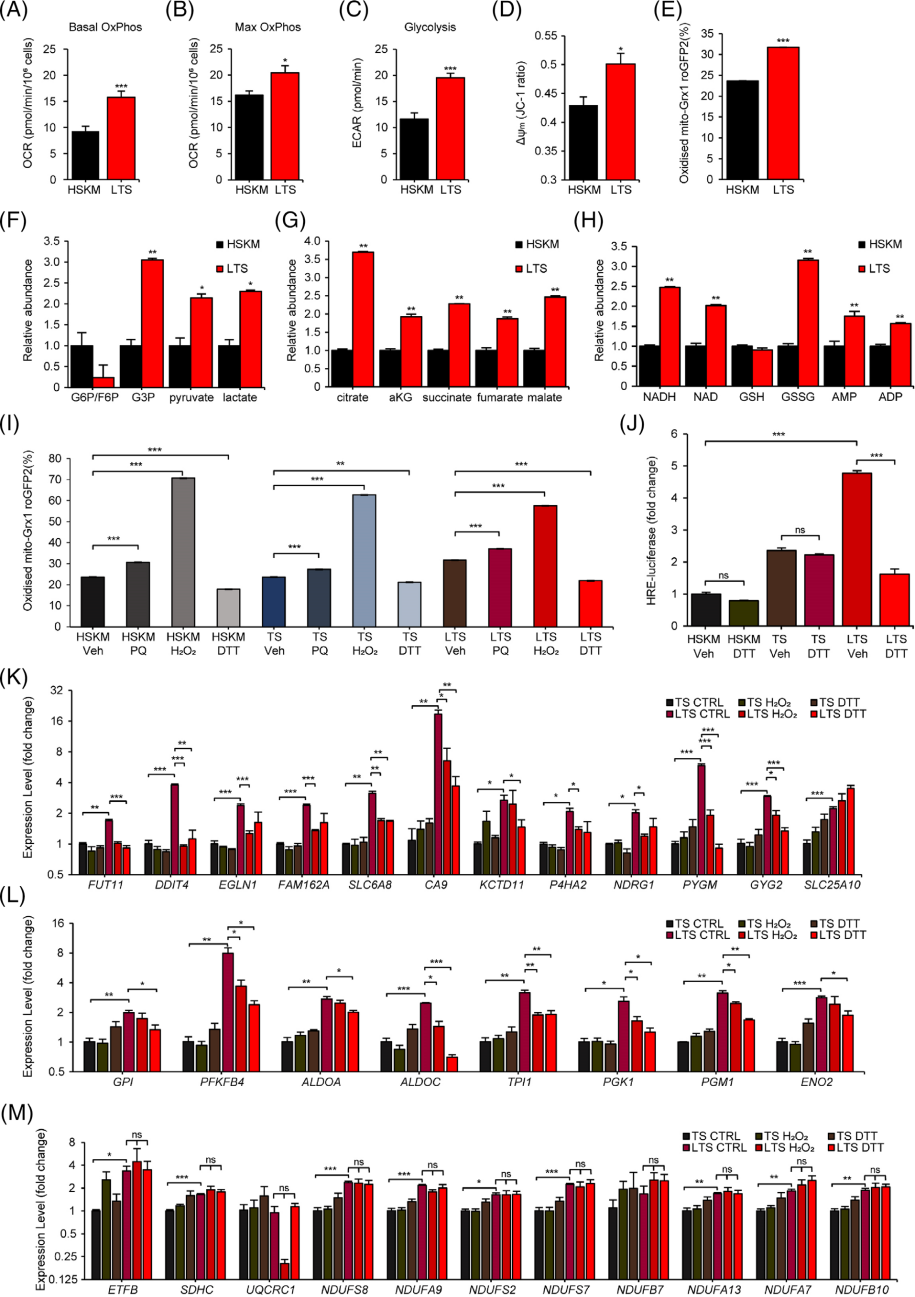
Metabolic effects of LIN28A in LIN28A, TERT, and sh‐p53 (LTS) muscle progenitors. (A–C). Seahorse analysis of basal and maximal OxPhos, glycolysis in LTS myoblasts, relative to young adult human skeletal muscle (HSKM) myoblasts (*N* = 3 wells of cells for each group). (D) Quantification of the mitochondrial membrane potential (Δψ_m_) in LTS myoblasts, relative to young adult HSKM myoblasts, according to the JC‐1 dye red:green fluorescence ratio (*N* = 3 wells of cells for each group). (E) Quantification of mitochondrial reactive oxygen species (mtROS) in LTS myoblasts, relative to young adult HSKM myoblasts, according to the mean percentage oxidation of the mito‐Grx1‐roGFP2 probe (*N* = 3 wells of cells for each group). (F–H) LC–MS/MS quantification of glycolysis metabolites, Krebs Cycle metabolites, redox, and bioenergetics‐related metabolites in LTS myoblasts (red) and adult HSKM myoblasts (black). G6P, glucose‐6‐phosphate. F6P, fructose‐6‐phosphate. G3P, glyceraldehyde‐3‐phosphate. aKG, α‐ketoglutarate; ADP, adenine diphosphate; AMP, adenine monophosphate; GSH, glutathionez; GSSG, glutathione disulfphide; NAD, nicotinamide adenine dinucleotide; NADH, reduced NAD (*N* = 3 wells of cells for each group). (I) Quantification of mtROS in young adult HSKM, TS, and LTS myoblasts, after treatment with the ROS‐modulating drugs paraquat (PQ), hydrogen peroxide (H_2_O_2_), dithiothreitol (DTT), or vehicle (Veh), according to the mean percentage oxidation of the mito‐Grx1‐roGFP2 probe (*N* = 3 wells of cells for each group). (J) Quantification of the HIF1α/hypoxia‐response element (HRE)‐luciferase reporter in young adult HSKM, TS, and LTS myoblasts, after treatment with DTT or vehicle control (*N* = 5 wells of cells for each group). (K) Quantitative RT‐PCR for hypoxia/HIF1α target mRNAs in LTS myoblasts, relative to TS myoblasts, after treatment with vehicle CTRL, H_2_O_2_, or DTT (*N* = 3 wells of cells for each group). (L) Quantitative RT‐PCR for glycolysis‐related mRNAs in LTS myoblasts, relative to TS myoblasts, after treatment with vehicle CTRL, H_2_O_2_, and DTT (*N* = 3 wells of cells for each group). (M) Quantitative RT‐PCR for OxPhos‐related mRNAs in LTS myoblasts, relative to TS myoblasts, after treatment with vehicle CTRL, H_2_O_2_, or DTT (*N* = 3 wells of cells for each group). *p < 0.05, **p < 0.01, ***p < 0.001.

To validate that OxPhos and glycolysis were indeed upregulated, we used the Seahorse extracellular flux analyser. Our analysis of oxygen consumption rates in the LTS progenitors revealed that LTS progenitors do indeed show significantly higher basal and maximal OxPhos rates (Figure 5A,B). Moreover, LTS progenitors also showed higher glycolysis rates (Figure 5C). We also found that the mitochondrial membrane potential Δψ_m_ in LTS progenitors was significantly increased (Figure 5D). In contrast, mitochondrial DNA and protein biogenesis did not increase, indicating that while the mitochondrial OxPhos activity levels were increased, total mitochondrial biogenesis did not increase (Figures S2D and S10). Since LIN28A has already been shown to bind OxPhos mRNAs to regulate OxPhos protein expression,^59^, ^60^, ^61^ it is plausible that LIN28A‐induced OxPhos would increase mtROS via an increased ETC flux and increased Δψ_m_. Previous studies had shown that a high mitochondrial membrane potential Δψ_m_ can shift the mtROS‐producing sites to a more reduced state, thereby increasing the propensity for mtROS production.^62^, ^63^ To validate this hypothesis, we used the mitochondrial Grx1‐roGFP2 reporter^64^ to determine mtROS levels in live cells. Our results showed that LTS progenitors had mildly but significantly higher mtROS than HSKM progenitors (Figure 5E). When we performed Liquid Chromatography‐tandem Mass Spectrometry (LC–MS/MS) metabolomics profiling, we confirmed that several glycolytic intermediates and mitochondrial Krebs Cycle intermediates were indeed increased in LTS progenitors (Figure 5F,G). LC–MS/MS metabolomics also showed that the glutathione disulfide/glutathione ratio, an indicator of oxidative stress, was higher in LTS progenitors (Figure 5H).

Since mtROS is well known to activate HIF1α, which in turn transactivates glycolysis genes, mtROS could also explain why the hypoxia and glycolysis signatures were upregulated.^63^, ^65^, ^66^, ^67^, ^68^, ^69^ If LIN28A was indeed specifically optimizing mtROS to prevent senescence, then ROS‐modulating drugs should have a disproportionately bigger effect on LTS progenitors than on TS or HSKM progenitors. To test this hypothesis, we subjected the various muscle progenitors to a variety of ROS‐modulating drugs and examined their effects on mtROS, gene expression, and proliferation rates. First, we found that hydrogen peroxide (H_2_O_2_) most reliably increased mtROS, whereas the cell‐permeable antioxidant dithiothreitol (DTT) most reliably reduced mtROS in all progenitor types (Figure 5I). Using a HIF1α/hypoxia‐response‐element luciferase reporter,^67^ we further validated that LIN28A does induce a HIF1α‐mediated hypoxic response in LTS progenitors relative to TS and HSKM progenitors, and that this stress response was dependent on ROS levels (Figure 5J).

When we subjected the progenitors to pro‐oxidant and antioxidant treatment, we found that both higher ROS and lower ROS could suppress the HIF1α targets (Figure 5K) and glycolysis genes (Figure 5L) specifically in LTS progenitors, but not in TS progenitors. This indicates that LIN28A was optimizing the mtROS level to upregulate HIF1α targets and glycolysis genes in LTS progenitors. In contrast, both H_2_O_2_ and DTT treatments failed to perturb the mildly higher OxPhos mRNA levels in LTS progenitors (Figure 5M), indicating that OxPhos mRNA regulation laid upstream of mtROS regulation. These results support a model whereby LIN28A ➔ optimal OxPhos and mtROS ➔ HIF1α‐driven hypoxic and glycolysis stress responses. We also used another antioxidant *N*‐acetyl‐L‐cysteine (NAC), to examine the above model. NAC treatment led to significant down‐regulation of some HIF1α targets and glycolysis genes specifically in LTS progenitors, more so than in TS progenitors (Figure S11A,B). NAC treatment also decreased the HIF1α/hypoxia‐response‐element luciferase reporter activity more drastically in LTS progenitors, than in TS or HSKM progenitors (Figure S11C), consistent with the model.

### 2.6 LIN28A‐mtROS‐HIF1α promotes self‐renewal

**FIGURE 6:**
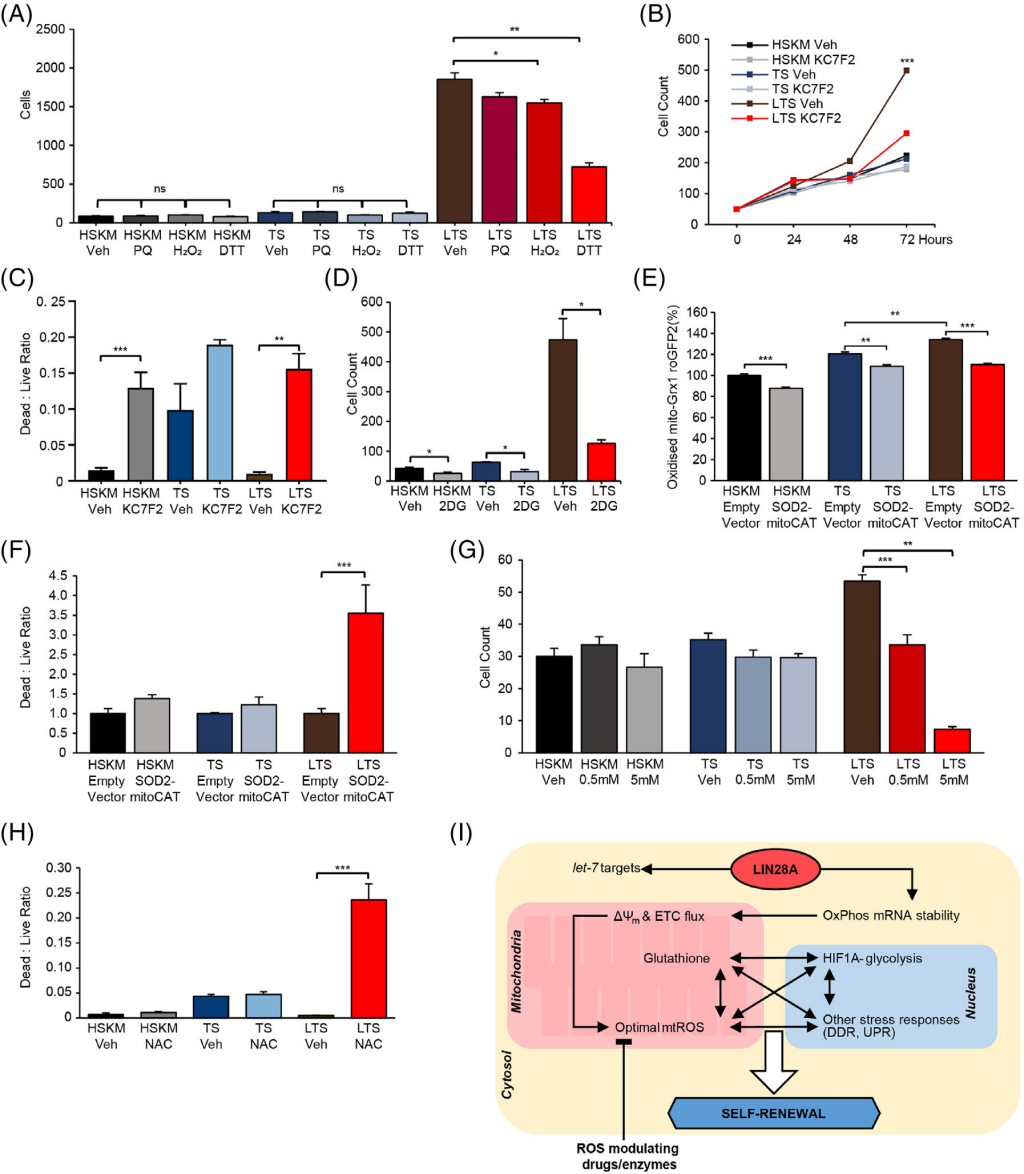
LIN28A‐mitochondrial reactive oxygen species (mtROS)‐HIF1α promotes self‐renewal of adult human muscle progenitors. (A) Final cell counts after treatment with vehicle control (Veh), hydrogen peroxide (H_2_O_2_), or dithiothreitol (DTT) titrations in equal initial numbers of TS and LIN28A, TERT, and sh‐p53 (LTS) myoblasts, relative to young adult human skeletal muscle (HSKM) myoblasts (*N* = 3 wells of cells for each group). (B) Cell counts over time after treatment with vehicle control (Veh) or the HIF1α inhibitor KC7F2 in equal initial numbers of TS and LTS myoblasts, relative to young adult HSKM myoblasts (*N* = 5 wells of cells for each group). (C) Ratio of dead to live cells, as measured by ethidium and calcein staining, after treatment with vehicle. control (Veh) or the HIF1α inhibitor KC7F2 in equal initial numbers of TS and LTS myoblasts, relative to young adult HSKM myoblasts (*N* = 5 wells of cells for each group). (D) Final cell counts after treatment with vehicle control or the glycolysis inhibitor 2‐deoxyglycose (2DG) in equal initial numbers of TS and LTS myoblasts, relative to young adult HSKM myoblasts (*N* = 5 wells of cells for each group). (E) Quantification of mtROS in HSKM, TS, and LTS myoblasts, before and after overexpression of mitochondria‐targeted superoxide dismutase 2 and mitochondria‐targeted catalase (SOD2‐mitoCAT), according to the mito‐Grx1‐roGFP2 probe (*N* = 5 wells of cells for each group). (F) Fold changes of ratio of dead to live cells after overexpression of SOD2‐mitoCAT relative to overexpression of the empty vector in HSKM, TS, and LTS myoblasts, respectively (*N* = 5 wells of cells for each group). (G) Final cell counts after treatment with vehicle control or 0.5–5 mM of the antioxidant *N*‐acetyl‐L‐cysteine (NAC) in equal initial numbers of HSKM, TS, and LTS myoblasts (*N* = 3 wells of cells for each group). (H) Ratio of dead to live cells, as measured by ethidium and calcein staining, after treatment with vehicle control (Veh) or 5 mM of the antioxidant NAC in equal initial numbers of TS and LTS myoblasts, relative to young adult HSKM myoblasts. (*N* = 3 wells of cells for each group). (I) Model of how LIN28A, mtROS, and the HIF1α‐associated metabolic stress response network promotes stem cell self‐renewal. *p < 0.05, **p < 0.01, ***p < 0.001; ns, not significant. UPR, unfolded protein response.

However, it remained unclear if these molecular changes were functionally responsible for LIN28A's effects on muscle progenitor self‐renewal. We tested and found that both pro‐oxidant and antioxidant treatments led to disproportionately more suppression of LTS progenitor proliferation, than of TS or HSKM progenitor proliferation (Figure 6A), supporting the model that mtROS optimization to promote proliferation is a mechanism specific to LIN28A + progenitors. Consistent with the model above, we also found that treatment with the HIF1α inhibitor (KC7F2) led to disproportionately more suppression of LTS progenitor proliferation, than of TS or HSKM progenitor proliferation (Figure 6B).

Live–dead staining of the cells revealed that in control vehicle‐treated cells, TS progenitors had more cell death than normal HSKM progenitors at baseline (Figure 6C), indicating that TS increased both proliferation and cell death, but LIN28A in LTS progenitors suppressed the increased cell death. Addition of the HIF1α inhibitor increased the proportion of dead cells similarly across HSKM, TS and LTS progenitors (Figure 6C), suggesting that LIN28A‐mediated suppression of cell death was over‐ridden by the HIF1α inhibitor, and that Lin28a promoted self‐renewal via HIF1α. The glycolysis inhibitor 2‐deoxyglucose (2DG) led to mild but significant decreases in all progenitors' proliferation, but the decrease was most pronounced in LTS progenitors (Figure 6D), again supporting the model that LIN28A is driving HIF1α and glycolysis to promote self‐renewal.

To further confirm the role of mtROS in this mechanistic model, we used genetic perturbation of mtROS‐specific enzymes to examine the necessity of mtROS to LIN28A's mechanism of action in cell proliferation. Targeted overexpression of mitochondrial superoxide dismutase 2 and mitochondrial catalase led to a significant decrease in mtROS (Figure 6E), more so in LTS progenitors. This decrease in mtROS translated to disproportionately lower cell viability in LTS progenitors than in TS or HSKM progenitors (Figure 6F). Treatment with NAC also further confirmed that the proliferation and survival of LTS progenitors were specifically more sensitive to antioxidants than TS or HSKM progenitors (Figure 6G,H).

Taken together, these results demonstrate that LIN28A specifically optimized mtROS‐HIF1α signalling and various stress response/repair pathways (Figure 6I) to promote the self‐renewal of human muscle progenitors. These results are consistent with the stress‐responsive signatures that we uncovered in the Lin28a + MuSCs in vivo (Preprint),^48^ and suggest that LIN28A activates molecular repair pathways in an evolutionarily conserved manner to maintain progenitors in a juvenile state. Strikingly, the mtROS induced a hypoxic stress response, glycolytic response, p53 response, and UPR to rejuvenate muscle progenitors (Figure 6I). In other contexts, such rejuvenative mtROS‐induced stress responses are collectively termed as mitohormesis.^70^

## 3 DISCUSSION

We have shown that pre‐senescent but old human muscle progenitors accumulate *let‐7* microRNAs, p53, p21^WAF1^, p16^INK4a^, and show telomere loss during ageing. A mini‐screen with embryonic factors revealed that a minimal set of LIN28A, telomerase, and shRNA‐mediated partial knockdown of p53 (LTS) was sufficient to significantly extend the lifespan of adult human muscle progenitors. We demonstrate that these rejuvenated muscle progenitors are endowed with extended self‐renewal and myogenic differentiation capacities, without oncogenic transformation into cancer cells. In fact, when aged and dysfunctional muscle progenitors from cancer cachexia patients were treated with LTS factors, their intrinsic self‐renewal and myogenic differentiation capacities were restored, and they could now contribute to the regeneration of muscles in vivo. Mechanistically, we found that *let‐7* microRNAs alone and their effects on IMPs, HMGA2, and associated IGF‐PI3K‐mTOR signalling^71^, ^72^ could not fully explain LIN28A's effects on muscle progenitors. LIN28A delayed the senescence of muscle progenitors by enhancing mitochondrial OxPhos, thereby optimizing mtROS and inducing a series of metabolic stress responses that promoted molecular repair and progenitor self‐renewal—a process known as mitohormesis in other contexts. Thus, reactivation of an embryonic metabolic programme with LIN28A, TERT, and partial blockade of p53 could delay the senescence phenotype of aged human progenitors, without activating the pluripotency factors nor reprogramming their lineage specification, and still restored their regenerative functions both in vitro and in vivo.

Few studies have investigated the role of LIN28A in human progenitors. Our and others' previous studies have shown that transgenic LIN28A can enhance foetal‐like morphogenesis and tissue regeneration in multiple mouse tissues, including the limb digits and skin,^60^ kidneys,^73^, ^74^ brain,^75^, ^76^ and retina.^77^ But, few studies have investigated its role in human cells, particularly in the skeletal muscles. Moreover, while previous studies have suggested Lin28a's role in regulating both glycolysis and OxPhos,^61^, ^72^, ^78^, ^79^ none have uncovered its importance in optimizing mtROS and mitohormesis to promote self‐renewal hitherto. Our mechanistic studies suggest that LIN28A fine‐tunes mtROS to optimize mitohormesis, and that both excessive upregulation or downregulation of ROS can disrupt LIN28A's beneficial effects.

Mitohormesis is defined as the coordinated compensatory response to mild mitochondrial stress that rapidly activates nucleocytoplasmic signalling pathways, and which ultimately alters gene expression to protect the cell against stressful perturbations and molecular damage.^70^ While the TS combo had little effect on mtROS and ultimately failed to enhance self‐renewal of muscle progenitors, the LTS combo upregulated mtROS to an optimal level and successfully delayed muscle progenitor senescence. These results support the emerging notion that, while excessive mtROS can cause a variety of pro‐ageing phenotypes in many contexts, mild mtROS can be beneficial at the right amounts in the right cell types.^63^ Interestingly recent studies have also shown that mouse embryonic myogenesis requires low but not excessive amounts of mtROS, tightly regulated by Pitx2/3.^80^, ^81^ Thus, our study validated the notion that mtROS and progenitor lifespan share a non‐linear relationship, and provided a genetic combo that optimizes mtROS to extend primary human progenitors' self‐renewal.

Our gene expression analyses showed that, in the context of LTS treatment, optimal levels of mtROS can robustly stimulate the HIF1α‐mediated hypoxic stress response against metabolic damage, XBP1S‐mediated UPR against protein damage, and p53‐mediated response against DNA damage (DDR, DNA Damage Response), all of which are already known to promote stem cell survival. It is interesting, but not surprising, that in the context of partial p53 knockdown, LIN28A‐mediated mtROS would restore some p53‐DDR activity.^63^ Previous studies had shown that the p53 gene network is tightly regulated and fine‐tuned through paradoxical signalling in progenitor cells.^37^ LIN28A‐mediated restoration of some p53 activity might be critical for ensuring proper muscle progenitor self‐renewal and differentiation, by preventing chromosomal instability and oncogenic transformation at the same time. Indeed, previous studies had shown that p53 is important in regulating myogenesis and many other lineage progenitors' proper differentiation.^40^, ^41^, ^42^ Besides the p53‐mediated DDR, the XBP1S‐mediated UPR proteotoxic stress response was also activated by LIN28A‐mediated mtROS. ROS‐mediated protein oxidation has long been known to cause proteotoxicity, and thus activate the UPR proteotoxic stress response.^82^ In fact, recent studies have shown that the UPR is needed for proper functioning of DNA repair enzymes, including the base excision repair pathway, to remove 8‐oxoguanine^83^, ^84^. Thus the UPR also cross‐talks with the DDR to pre‐emptively protect progenitors against genotoxic stress. Another pathway stimulated by UPR is autophagy,^85^ which was previously shown to be important in rescuing geriatric mouse muscle stem cells from senescence.^86^ Another mitohormetic pathway that cross‐talks with the p53‐mediated DNA damage response is the HIF1α‐mediated hypoxic stress response.^68^ Multiple previous studies had already shown that ROS activates HIF1α to drive the hypoxic stress response.^63^, ^68^ Moreover, it is well‐known that HIF1α can transactivate glycolysis genes.^68^ Studies have shown that the hypoxic stress response is important for mammalian regeneration and the biology of naked mole rats,^87^, ^88^ which are remarkably resistant to cancer and ageing for their size, suggesting that the hypoxic stress response could be an important arm of mitohormesis in mammalian rejuvenation.

Interestingly, ROS can also directly stimulate glycolytic flux, especially into the pentose phosphate pathway (PPP^89^). Glycolysis intermediates such as fructose‐6‐phosphate can be diverted into the PPP to fuel NADPH synthesis for reducing oxidative stress, and nucleotide synthesis for stem cell proliferation. Other glycolytic intermediates like 3‐phosphoglycerate and pyruvate can be diverted into amino acid synthesis, whereas dihydroxyacetone‐phosphate and acetyl‐CoA can be diverted into lipid synthesis for anabolic growth.^90^ NADPH and amino acid synthesis are particularly important for synthesizing glutathione in the context of an oxidative stress response, which is also activated by the UPR (Ron and Walter, 2007). This is attested by the rise in the total pool of oxidized and reduced glutathione in LTS progenitors, compared with HSKM progenitors. Both NAD+ and NADH were also upregulated by LTS, but there were no significant changes in the NAD+/NADH ratio, suggesting that while changes in NAD+ and Sirt1 activity are both crucial for regulating muscle stem cell self‐renewal and muscle regeneration,^61^, ^91^ NAD+ and Sirt1 are less likely to be responsible for LTS' effects in this context. Nevertheless, the mutually reinforcing nature of these protective metabolic pathways suggests that mitohormesis is a conserved and well‐coordinated programme of stress‐responsive pathways to control progenitor lifespan.

These mitohormetic stress response mechanisms might also shed more light on why LIN28A alone is insufficient to induce oncogenic transformation or induced pluripotent stem cell reprogramming on its own, but rather facilitates progenitor self‐renewal instead. It should also be noted that both *Lin28* ^KO^ and Tert^KO^ cells can still undergo tumour initiation through other pathways, that is, they are not as critical for cancer as they are critically needed for normal development.^45^, ^92^ Prior research has demonstrated that telomere shortening or telomerase inhibition is more toxic to normal stem cells, than cancer cells,^92^, ^93^ which has led to the failure of such approaches clinically. In fact, TERT deficiency promotes (not prevent) malignant transformation by accelerating senescence and SASP and chromosomal instability, whereas a transient upregulation of TERT delays senescence and prevents transformation.^94^, ^95^

It is also interesting that LIN28A induced some level of dedifferentiation to produce PAX3^high^ skeletal muscle progenitors. Genetic loss‐of‐function studies have demonstrated that embryonic Pax3+ progenitors are essential to generate all the myogenic cells in the limb, including all embryonic, foetal, and adult myoblasts and myofibres.^3^, ^23^, ^24^, ^25^ Interestingly, some adult MuSCs have also been reported to express Pax3 to regulate regenerative capacity^3^, ^96^, ^97^, ^98^. Thus, LIN28A could dedifferentiate PAX3^low^ myoblasts into PAX3^high^ muscle progenitors with an increased ability to engraft and regenerate in injured skeletal muscles.

In summary, our studies indicate that aged muscle progenitors can be delayed from entering the senescence state by inducing embryonic programmes that activate mitohormesis. Future work could focus on therapies that transiently reactivate such embryonic metabolic programmes in tissue progenitors ex vivo, instead of permanent transgenesis, given concerns on viral transgenesis and that senescence does play important roles in normal wound healing and tumour suppression.^99^ Such therapies could complement current efforts to employ senolytic therapies and cell therapies to restore our innate regenerative capacities,^100^, ^101^, ^102^, ^103^, ^104^ with implications for regenerative medicines in ageing‐related diseases such as cachexia and sarcopenia in future.

## 4 MATERIALS AND METHODS

### 4.1 Cell culture and differentiation

Young adult primary HSKM progenitors isolated from a 20‐year‐old female subject's quadriceps muscles were seeded onto plates coated with 0.1% gelatin solution (Merck‐Millipore) and incubated at 37°C, 5% CO_2_ with growth medium, comprising of Dulbeccos Modified Eagle Medium (DMEM)/F‐12 (Gibco) with 20% foetal bovine serum (FBS; GE Healthcare), 1% L‐glutamine (Gibco) and 1% penicillin‐streptomycin (Gibco). At each passage, after reaching 80% confluence, cells were trypsinized and diluted 1:4. Differentiation was initiated by replacing growth media with differentiation medium, comprising of DMEM/F‐12, 2% KnockOut Serum Replacement (Gibco), 1% L‐glutamine (Gibco) and 1% penicillin‐streptomycin (Gibco), when the young adult HSKM progenitors were 80%–100% confluent. These young adult HSKM progenitors were previously validated to be 100% MYOD1^+^ during the proliferative stage, with robust expression of myogenic markers after differentiation.^46^

### 4.2 Virus production

The following plasmids were used to produce viruses for the various transgenic cell lines: lentiviral plasmid (Addgene no. 19119), dR8.2 packaging plasmid (Addgene no. 8455), VSV‐G envelope plasmid (Addgene no. 8454), pMSCV‐mLin28A (Addgene no. 26357), pBABE‐hygro‐hTERT (Addgene no. 1773), shp53 pLKO.1 puro (Addgene no. 19119), pLenti CMV GFP Blast (659‐1; Addgene no. 17445), pLPCX mito Grx1‐roGFP2 (Addgene no. 64977), pLenti X2 Blast/shp16 (w112‐1; Addgene no. 22261), pLKO‐RB1‐shRNA19 (Addgene no. 25640), hypoxia‐response element‐luciferase (Addgene no. 26731), and pLKO.1‐hPGK‐Neo (SHCLND‐NM_058197, TRCN0000265840, Sigma‐Aldrich). Viral supernatants were collected within the 48‐h to 96‐h window and filtered with a 0.45 μm filter (Sartorius). Virally transduced cells of the relevant types were selected with the relevant antibiotics for 3 days, and the transduction efficiency was typically about 80%.

### 4.3 Cachexia patient cell lines

Aged primary HSKM progenitors were derived from the rectus abdominus of two aged patient donors with cachexia (age/weight: 80 years/55 kg and 84 years/44 kg) during tumour‐resection surgery. Procedures were performed in accordance to ethical legislation and SingHealth Institutional Review Board guidelines. Muscle progenitors were isolated according to previously published protocols.^105^ Cells were maintained in culture medium and differentiated with differentiation medium as described above.

### 4.4 Population doubling curve

1.5 × 10^4^ cells were seeded in one gelatin‐coated well of a 6‐well plate (Falcon) with growth medium comprising of DMEM/F‐12 (Gibco) with 20% FBS (GE), 1% L‐glutamine (Gibco) and 1% penicillin‐streptomycin (Gibco). Upon reaching a confluency of 80%–100%, cells were lifted with 0.25% trypsin (Gibco) and counted, 1.5 × 10^4^ cells were then subcultured. This process was repeated until cells could no longer achieve 80% confluency, or until a period of 100 days. Recorded cell counts were calculated as cumulative population doubling levels and plotted over the number of days in culture.

### 4.5 Quantitative PCR

RNA was extracted by TRIzol (Thermo Fisher) and reverse transcribed with Superscript III (Thermo Fisher) according to manufacturer's instructions. The resulting cDNA was diluted 5× before performing qPCR with KAPA SYBR FAST on ABI Prism 7900HT (Applied Biosystems) according to manufacturers' instructions.

### 4.6 Western blots

Protein was extracted with RIPA buffer (Thermo Fisher) supplemented with protease inhibitor cocktails I and II (Sigma) and phosphatase inhibitor cocktail set III (Calbiochem). Protein was quantified with Pierce BCA protein assay kit (Thermo Fisher) and analysed with a Sunrise Tecan plate reader. After Sodium dodecyl sulfate‐polyacrylamide gel electrophoresis and electro‐transfer onto Polyvinyl‐Difluor membranes (GE Healthcare), Western blotting was performed with the following primary antibodies and concentrations: GAPDH (sc‐365062; Santa Cruz; 1:10,000), PAX3 (AB_528426; DSHB; 0.5 μg/mL), Twist2 (ab66031; Abcam; 1 μg/mL), IMP1/2/3 (sc‐271785; Santa Cruz; 1:1000), HMGA2 (no. 5269S; Cell Signalling; 1:1000), tubulin (ab210797; Abcam; 1:1000), p53 (sc‐126; Santa Cruz; 1:100), citrate synthase (G‐3; sc‐390693; Santa Cruz; 1:1000). Blots were stained with secondary antibodies conjugated with Horseradish Peroxidase, 1:2500, (Promega W401B, W402B) and visualized with ECL prime western blotting detection reagent kit (GE Healthcare).

### 4.7 Immunofluorescence

Cells were first washed with Phosphate Buffer Saline (Thermo Fisher) and fixed with 4% Paraformaldehyde (MS). Cells were stained with the following primary antibodies and concentrations, MYHC‐IIb eFluor 660 (50‐6503‐32; Thermo Fisher; 1:100), α‐actinin (sc‐7453; Santa Cruz; 1:500), and myogenin (sc‐576; Santa Cruz; 1:200). Anti‐PARP‐1 (Santa, SC‐8007), anti‐XRCC1 (Cell Signalling, 2735S), anti‐gamma H2AX (Abcam, 2893), anti‐53BP1 (Abcam, ab175933), and anti‐Osteocalcin (Santa, SC‐365797).The following secondary antibodies were also used together with non‐conjugated primary antibodies, Goat‐anti‐mouse Alexa Fluor 488 (A11001; Thermo Fisher; 1:500), Goat‐anti‐rabbit Alexa Fluor 594 (A11012; Thermo Fisher; 1:500), and Goat‐anti‐mouse Alexa Fluor 647 (A21235; Thermo Fisher; 1:500). 4'6‐Diamidino‐2‐Phenylindole (d9542; Sigma) was used as a nuclear counter stain according to manufacturer's recommendations. Stained cells were imaged with a Zeiss fluorescence microscope.

### 4.8 miRNA quantitative PCR

A 500 ng of total RNA extracted by TRIzol (Thermo Fisher) was amplified by miScript RT Kit (Qiagen). With the resulting cDNA libraries, real‐time quantitative PCR was performed using the miScript SYBR Green PCR kit (Qiagen) on ABI Prism 7900HT (Applied Biosystems) according to manufacturers' instructions. The following miScript Primer Assays (Qiagen) were used in RT‐qPCR, Hs_let‐7a_2 (MS00031220), Hs_let‐7b_1 (MS00003122), Hs_let‐7e_3 (MS00031227), Hs_let‐7g_2 (MS00008337), Hs_RNU6‐2_11 (MS00033740), and Hs_SNORD61_11 (MS00033705).

### 4.9 Illumina transcriptomics and analysis

TRIzol (Thermo Fisher) was used to extract total RNA and purified by ethanol precipitation. cRNA libraries were generated with Illumina TotalPrep RNA Amplification Kit (Thermo Fisher) and hybridized to HumanHT‐12 v4.0 BeadChips (Illumina) according to manufacturer's protocol. Beadchips were scanned with a Bead Array Reader (Illumina) and results were extracted with Illumina BeadScan and Genome Studio. Further analysis was performed with GSEA (Broad Institute). PCA of the dataset was calculated in R version 3.1.2 using the *prcomp* function of the *stats* package.

### 4.10 Extracellular flux and oxygen consumption measurements

Cells were seeded onto Seahorse XFe96 cell culture plate (Agilent) at a density of 6000–8000 cells/well with culture medium for 24 h. Subsequently, media was changed to Seahorse minimal media and incubated in a non‐CO_2_, 37°C incubator for 1 h as per manufacturer's instructions. Utility plate was loaded with the following nutrient/drugs, Port A—10 mM glucose (for glycolysis) or 10 mM pyruvate (for OxPhos); Port B—1 μM oligomycin; Port C—0.5 μM FCCP; Port D—0.5 μM Antimycin A. Assay was run on a Seahorse XF extracellular flux analyser (Agilent) according to manufacturer's protocols.

### 4.11 SA‐β‐gal assays

SA‐β‐gal activity was determined with the Senescence Cells Histochemical Staining Kit (Sigma‐Aldrich) according to manufacturer's protocols. Six representative images were captured on a TS100 inverted light microscope (Nikon) and camera module. For each cell line, cells stained blue by cleaved X‐gal, indicating the presence of SA‐β‐gal, were counted as a percentage of the total number of cells in all images.

### 4.12 Cytogenetics

Cells were seeded in gelatin‐coated 6‐well plates and cultured up to a maximum of 50% confluency to avoid myoblast fusion. Cells were first treated with colcemid and 5‐Bromo‐2‐DeoxyUridine overnight before harvesting with EDTA. A fixative solution (1:3 = glacial acetic acid: methanol) was used to fix pelleted cells prior to slide preparation, Giemsa banding, and mounting. Twenty metaphase spreads were prepared for each cell line for detailed analyses and karyotyping.

### 4.13 *let‐7* microRNA mimic

HSKM, TS and LTS cells were seeded in gelatin‐coated 6‐well plates at a density of 4 × 10^4^ cells/well and maintained with culture medium. Polyetherimide (14.5 ng/μL), mature Mirvana hsa‐let‐7a‐5p (no. 4464066; Assay ID: MC10050; Thermo Fisher) and/or hsa‐let‐7b‐5p (no. 4464066; Assay ID: MC11050; Thermo Fisher) mimic (0.25 μM) or Cy5‐conjugated scramble RNAi control, was mixed with serum‐free DMEM to a total volume of 200 μL. This mixture was transfected onto each well.

### 4.14 KC7F2, ROS modulators, and 2‐DG

HSKM, TS and LTS cells were seeded in gelatin‐coated 6‐well plates at a density of 4 × 10^4^ cells/well and maintained with culture medium. One day after seeding, culture medium was replaced with culture medium containing either paraquat (Sigma‐Aldrich; 2 μM), hydrogen peroxide (ICM Pharma; 100 μM), DTT (Thermo Fisher; 250 μM), NAC (Sigma‐Aldrich; 0.5–5 mM), 2‐DG (Sigma‐Aldrich; 2.5 mM), KC7F2 (Sigma‐Aldrich; 10 μM), or Dimethyl Sulfoxide/H_2_O controls. For ROS modulators, cells were allowed to grow for 72 h post‐treatment, whereas for 2‐DG, cells were allowed to grow for 8 days post‐treatment.

### 4.15 Flow cytometry oxidation state assay with mito‐Grx1‐roGFP2

Retroviruses were created with the pLPCX mito Grx1‐roGFP2 plasmid according to the virus production subsection. HSKM, TS, and LTS cells were transduced with the roGFP2 retrovirus for 48 h. Cells were allowed to expand to 15‐cm tissue culture plates and then harvested for fluorescence‐activated cell sorting for roGFP2+ cells using the FITC channel with a BD FACS Aria II cell sorter. HSKM, TS, and LTS cells were seeded in gelatin‐coated 6‐well plates at a density of 4 × 104 cells/well and maintained with culture medium. One day after seeding, culture medium was replaced with culture medium containing either paraquat (Sigma‐Aldrich; 2 μM), hydrogen peroxide (ICM Pharma; 100 μM), or DTT (Thermo Fisher; 250 μM). Cells were allowed to grow for 48 h and then harvested for flow cytometry using a BD LSR Fortessa x‐20 analyser. HSKM, TS, and LTS cells were used as non‐fluorescent gating controls for each of their respective roGFP2+ counterparts. For each cell line, FSC, SSC, 405 nm/AmCyan, and 488 nm/FITC channels were captured. FCS files were exported from FACS Diva and imported into FlowJo, where raw‐value CSV files were exported. Raw values of 405/488 nm ratio were calculated and then averaged for each cell line and condition. The % oxidized value was calculated according previously published methods,^106^ where the difference between the 405/488 value of the vehicle control and DTT‐treated condition was divided by the difference between the 405/488 value of the hydrogen peroxide and DTT‐treated conditions.

### 4.16 Statistics and reproducibility

All statistical analyses were performed using GraphPad Prism 6 (GraphPad Software). Data are presented as mean ± SEM. Differences between groups were tested for statistical significance by using the two‐sample *t*‐test. A *p* < 0.05 was considered significant. The number of biological (non‐technical) replicates for each experiment is indicated in the legends.

## AUTHOR CONTRIBUTIONS

Peng Wang, Xupeng Liu, Yu Chen, Jun‐Hao Elwin Tan, and Ng Shyh‐Chang designed all experiments. Peng Wang, Xupeng Liu, Yu Chen, Jun‐Hao Elwin Tan, Min‐Wen Jason Chua, Yan‐Jiang Benjamin Chua performed in vitro and in vivo experiments and collected data. Min‐Wen Jason Chua designed, performed, and analysed in vivo engraftment studies. Ziyue Yao, Luyao Guo, and Dan Song. provided technical assistance. Lanfang Luo helped with cryosection and immunofluorescence experiments. Shilin Ma helped with western blot experiments. Wenwu Ma helped with bioinformatics analysis. Ng Shyh‐Chang designed and supervised the overall project.

## FUNDING INFORMATION

This work was supported by the Strategic Priority Research Program of the CAS (XDA16010109), the National Natural Science Foundation of China (91957202), the National Key R&D Program of China (2019YFA0801700), and the Key Research Program of the CAS (KJZD‐SW‐L04). Ng Shyh‐Chang is also a Howard Hughes Medical Institute (HHMI) International Scholar.

## CONFLICT OF INTEREST STATEMENT

The authors have no conflicts of interests to declare.

## Supporting information


**Data S1:** Supporting information.

## Data Availability

The data that support the findings of this study are available from the corresponding author upon reasonable request.
