# Supplementary material for: Adult progenitor rejuvenation with embryonic factors
Source: Cell Prolif. 2023 May 12;56(5):e13459. doi: 10.1111/cpr.13459 (PMC10212697; doi:10.1111/cpr.13459)
Supplement: Supplementary file 1 — Data S1: Supporting information. [file CPR-56-e13459-s001.docx]

**Supplemental Figures**

**
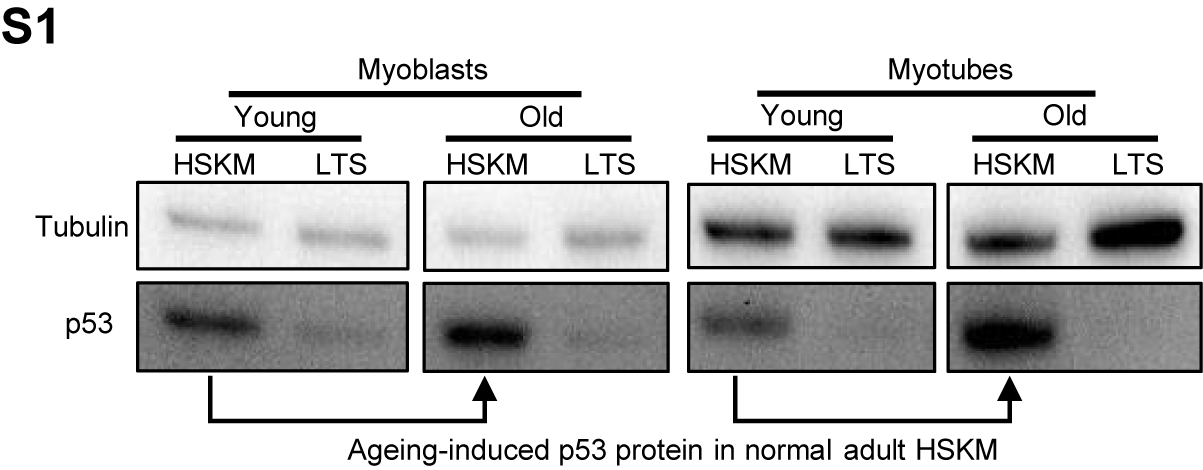
**

**Figure S1. Expression of p53 protein during muscle progenitor ageing and differentiation.**

Western blot for p53 protein, relative to tubulin protein, in young and old adult HSKM and LTS myoblasts and myotubes. The quantification of WB bands are shown in Figure S2.


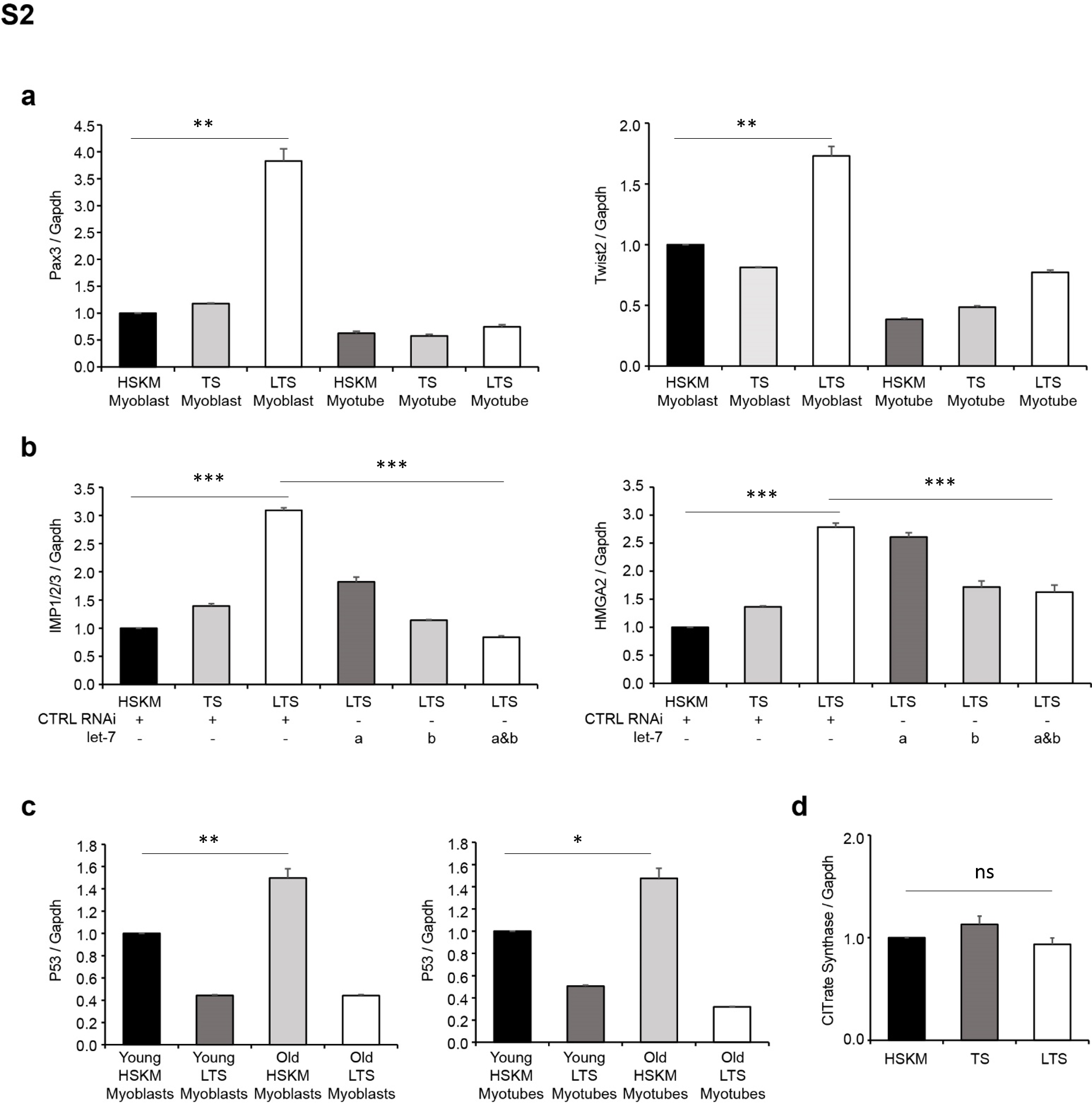


**Figure S2. The quantification of WB bands in Fig 3e, 4d, S1, S8b.**

(a). The protein expression of Pax3 and Twist2 were quantified by bands’ intensities ratio of Pax3/Gapdh and Twist2/Gapdh.

(b). The protein expression of IMP1/2/3 and HMGA2 were quantified by bands’ intensities ratio of IMP1/2/3 /Gapdh and HMGA2 /Gapdh.

(c). The protein expression of P53 was quantified by bands’ intensities ratio of P53/Gapdh.

(d). The protein expression of Citrate Synthase was quantified by bands’ intensities ratio of Citrate Synthase /Gapdh.

* P < 0.05, ** P < 0.01,*** P < 0.001,ns not significant.

**
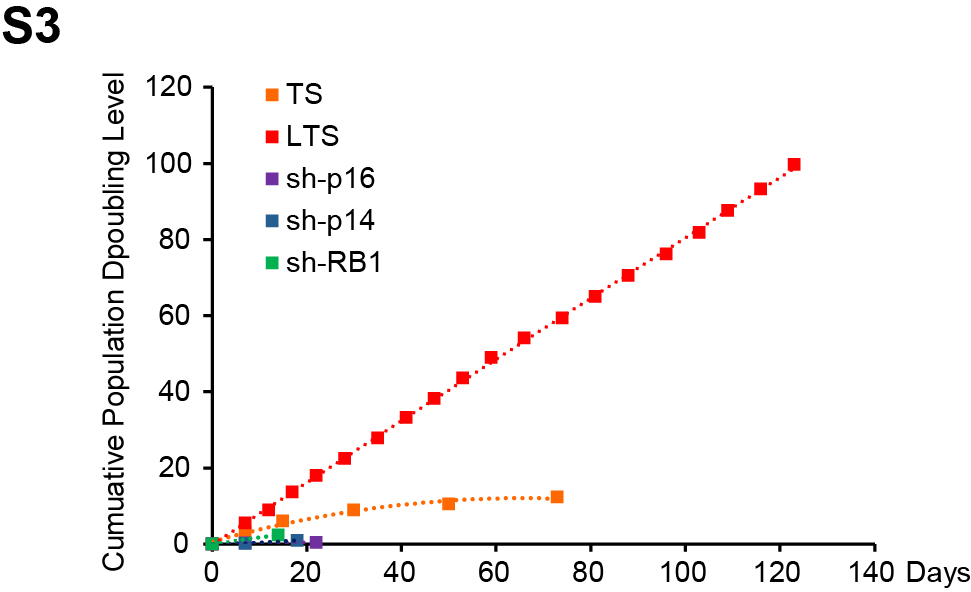
**

**Figure S3.** **Population doubling curves for young HSKM and other transgenic myoblasts.**

Young HSKM myoblasts (black), and young adult HSKM myoblasts transduced with hTERT and shp53 (TS, orange), or LIN28A hTERT and shp53 (LTS, red) showed an increasing order of proliferation rate. Lentiviral shRNA knockdowns of p16^INK4a^ (sh-p16, purple), p14^Arf^ (sh-p14, blue), and RB1 (sh-RB1, green) were also attempted, but were not followed up upon as they appeared to be prematurely senescent.

**
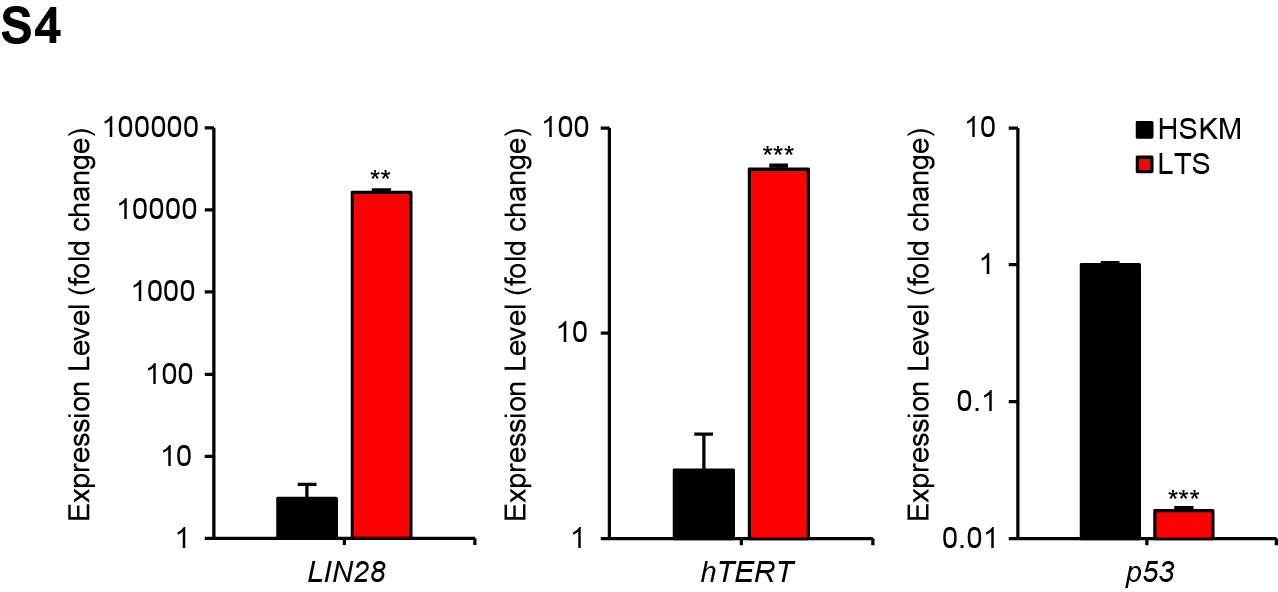
**

**Figure S4.** **Expression levels of the LTS factors.**

Quantitative RT-PCR for mRNAs of Lin28, hTERT, and p53 in LTS myoblasts, relative to young adult HSKM myoblasts. N=3 wells of cells for each group.** P < 0.01, *** P < 0.001


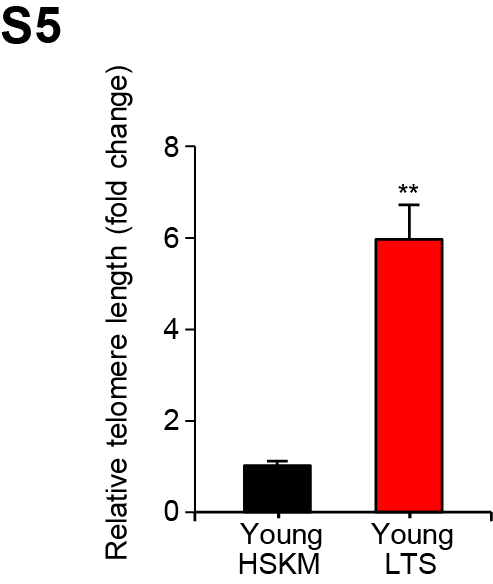


**Figure S5.** **Effects of the LTS factors on telomere length.**

Quantitative RT-PCR for telomere length in young LTS myoblasts, relative to young adult HSKM myoblasts. N=5 wells of cells.** P < 0.01.


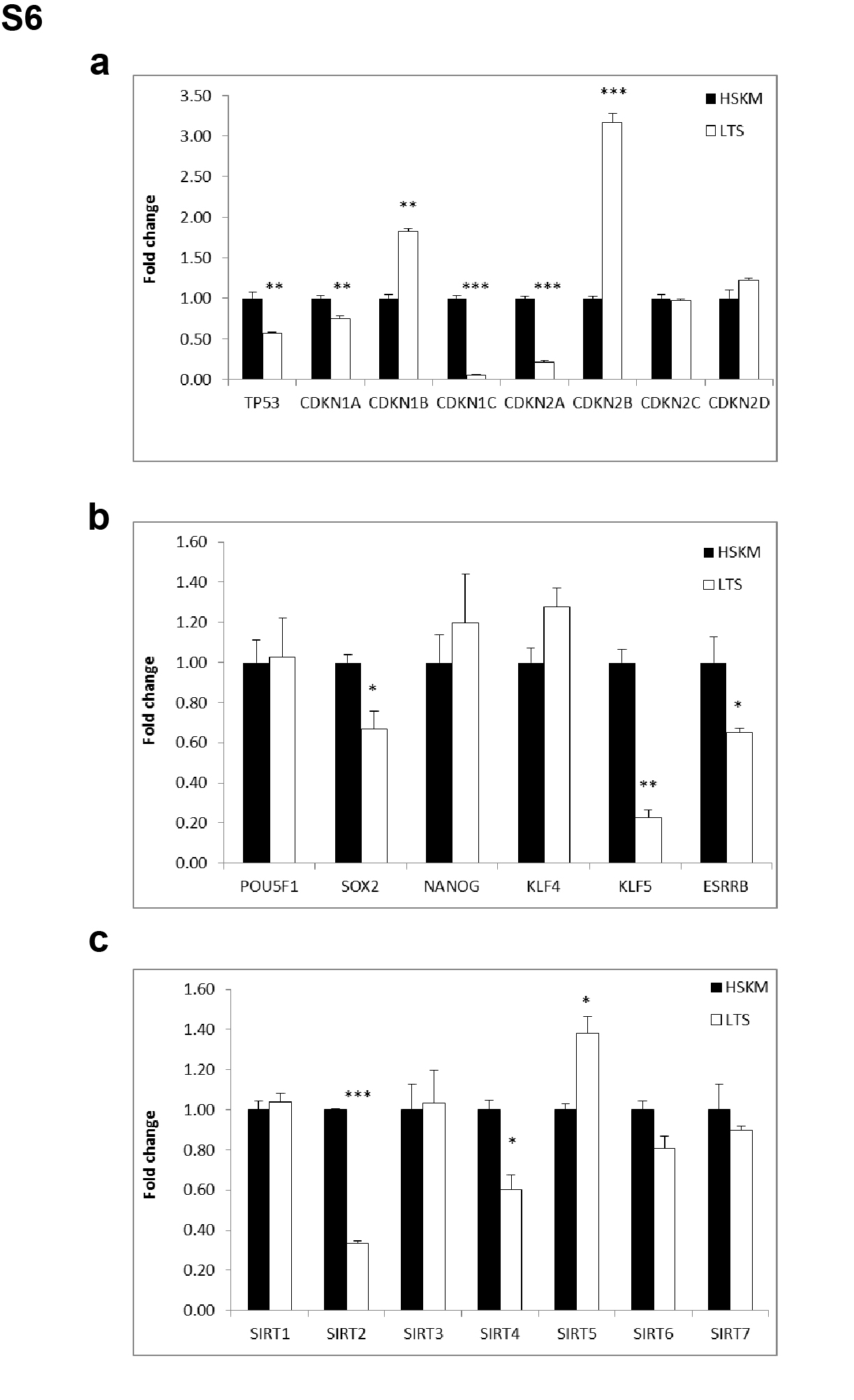


**Figure S6. The gene expression level of senescence markers, pluripotency markers and the SIRT family in young HSKM vs LTS.**

a. The gene expression level of senescence markers in young HSKM vs LTS.

b. The gene expression level of pluripotency markers in young HSKM vs LTS.

c. The gene expression level of the SIRT family in young HSKM vs LTS.

N=3 wells of cells for each group. * P < 0.05, ** P < 0.01, *** P < 0.001.


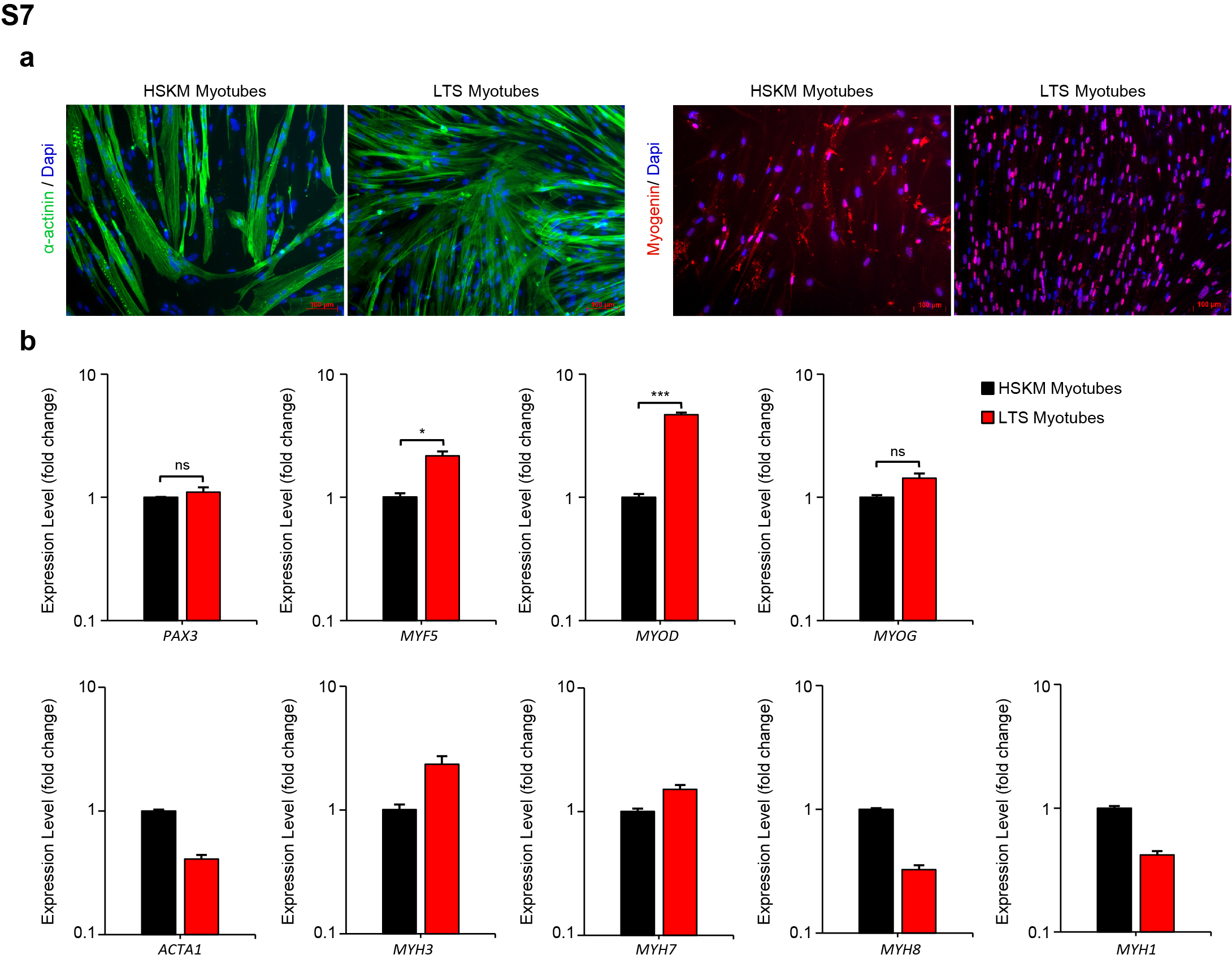


**Figure S7.** **Effects of the LTS factors on muscle progenitor differentiation.**

a. Immunofluorescence staining for the myotube protein markers α-actinin (green) and nuclear myogenin (red) in young HSKM myotubes, relative to LTS myotubes. Cells were counterstained with DAPI (blue) to visualize the myonuclei. Scale bars 100 µm.

b. Quantitative RT-PCR for *PAX3, MYF5, MYOD, MYOG* and the terminal differentiation markers *MYH1, MYH3, MYH8, MYH7*, *ACTA1* in LTS myotubes, relative to young adult HSKM myotubes. N=5 wells of cells for each group.


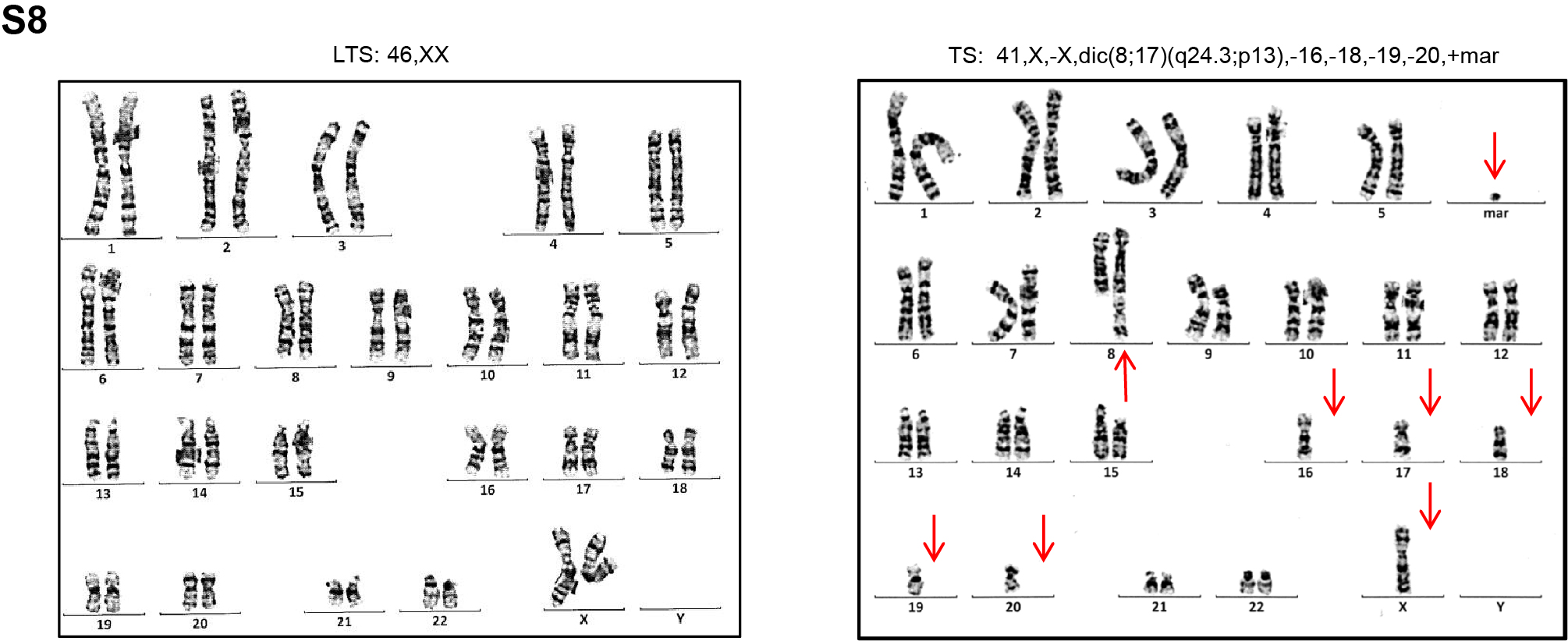


**Figure S8. Representative karyotype analysis of old LTS and TS progenitors.**

Representative karyotype analysis of old LTS and TS progenitors, which are [46,XX] and [41,X, X,dic(8;17)(q24.3;p13),-16,-18,-19,-20,+mar], respectively. Chromosomal aberrations are indicated with red arrows.


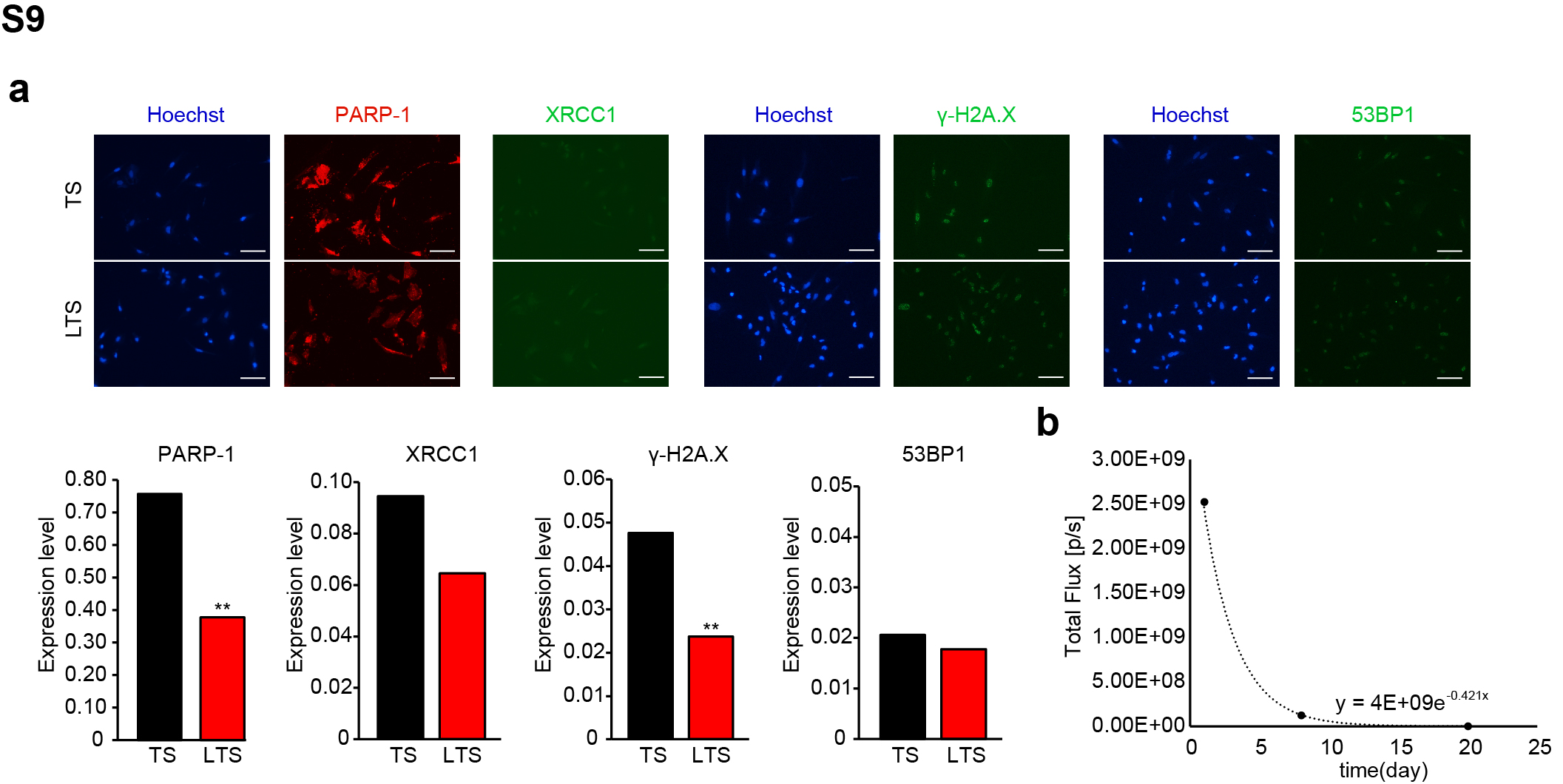


**Figure S9. Genomic stability and tumorigenicity of LTS progenitors.**

**a.** Quantification for the expression level of PARP-1, XRCC1, γ-H2A.X and 53BP1 through immunofluorescence in TS and LTS myoblasts. Scale bars 100 µm. N=5 wells of cells for each group.

**b**. Investigation of the tumorigenic potential of LTS cells. After LTS cells were lentivirally infected with luciferase, they were subcutaneously transplanted with Matrigel into NSG mice and we monitored transplant engraftment in vivo by bioluminescence imaging for 3 weeks. The subcutaneous graft disappeared by 2 weeks.

N=3 mice.


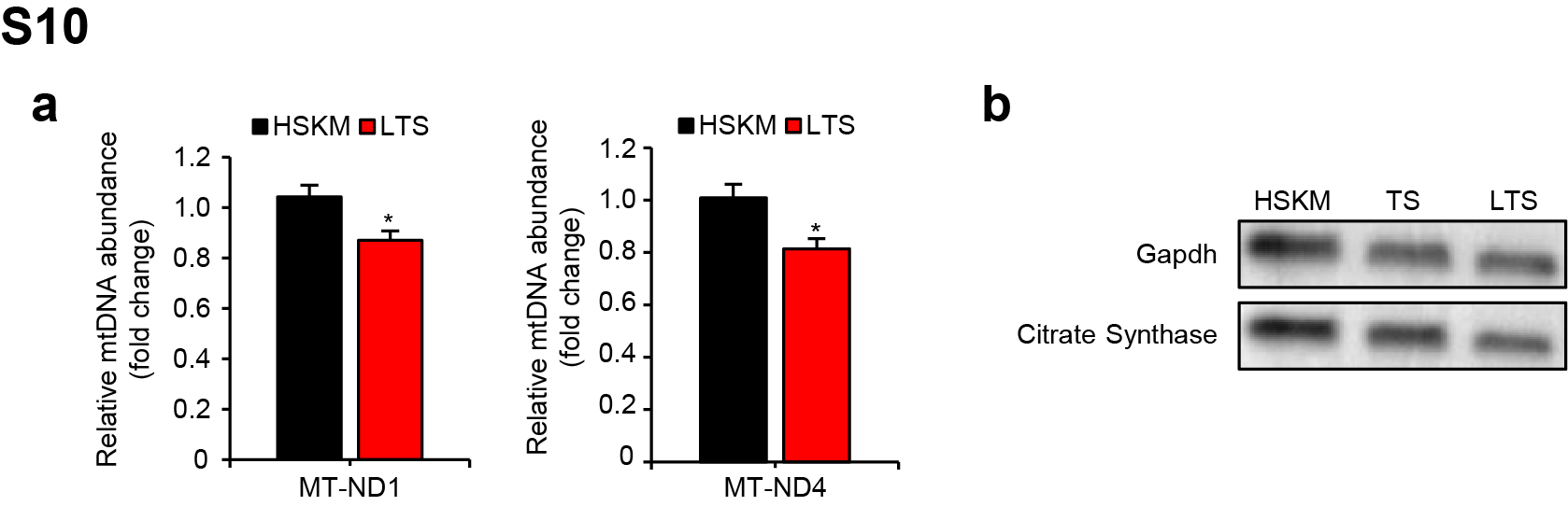


**Figure S10.** **Effects of the LTS factors on mitochondrial DNA (mtDNA) abundance and mitochondrial biogenesis.**

a. Quantitative RT-PCR for mitochondrial NADH-ubiquinone oxidoreductase chain 1 (*MT-ND1*) and 4 (*MT-ND4*), relative to the nuclear reference gene *B2M*, to assess mtDNA copy number in adult HSKM myoblasts, relative to LTS myoblasts. N=3 wells of cells for each group.* P < 0.05.

b. Western blot for mitochondrial citrate synthase protein, relative to GAPDH protein, in TS and LTS myoblasts, relative to adult HSKM myoblasts. The quantification of WB bands are shown in Figure S2.


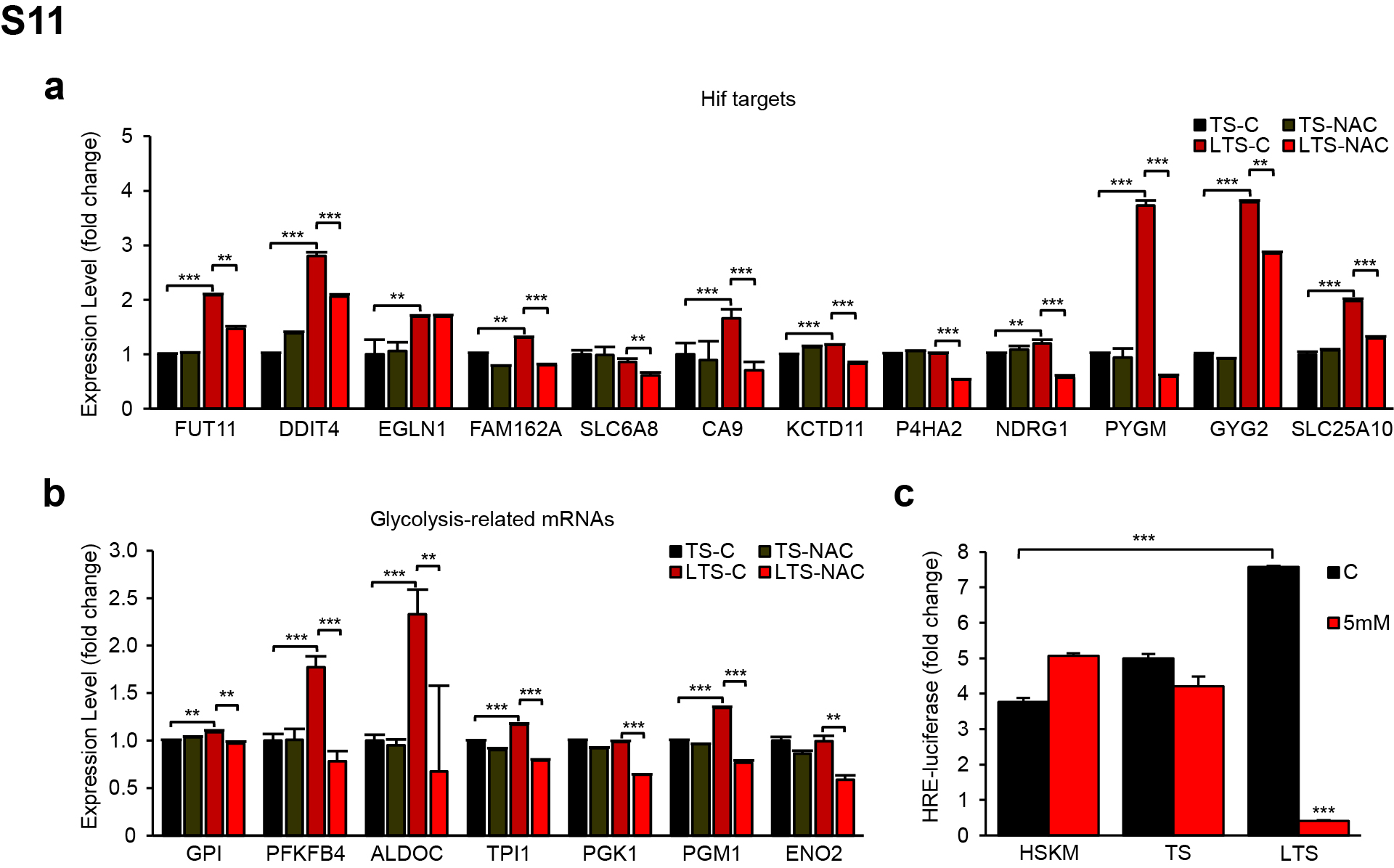


**Figure S11. Metabolic effects of LIN28A in LTS muscle progenitors.**

(**a**) Quantitative RT-PCR for hypoxia/HIF1α target mRNAs in LTS myoblasts, relative to TS myoblasts, after treatment with vehicle control (CTRL), N-acetyl-L-cysteine (NAC). N=3 wells of cells for each group.

(**b**) Quantitative RT-PCR for glycolysis-related mRNAs in LTS myoblasts, relative to TS myoblasts, after treatment with vehicle control (CTRL), N-acetyl-L-cysteine (NAC). N=3 wells of cells for each group.

(**c**) Quantification of the HIF1α/hypoxia-response element (HRE)-luciferase reporter in young adult HSKM, TS and LTS myoblasts, after treatment with 5mM of the antioxidant N-acetyl-L-cysteine (NAC) or vehicle control. N=5 wells of cells for each group.

* P < 0.05, ** P < 0.01, *** P < 0.001.
